# Data-driven spectral analysis for coordinative structures in periodic human locomotion

**DOI:** 10.1038/s41598-019-53187-1

**Published:** 2019-11-14

**Authors:** Keisuke Fujii, Naoya Takeishi, Benio Kibushi, Motoki Kouzaki, Yoshinobu Kawahara

**Affiliations:** 1 Graduate School of Informatics, Nagoya University, Nagoya, Japan; 2 RIKEN Center for Advanced Intelligence Project, Tokyo, Japan; 3 School of Sport Sciences, Waseda University, Tokyo, Japan; 4 Graduate School of Human and Environmental Studies, Kyoto University, Kyoto, Japan; 5 Institute of Mathematics for Industry, Kyushu University, Fukuoka, Japan

**Keywords:** Machine learning, Dynamical systems

## Abstract

Living organisms dynamically and flexibly operate a great number of components. As one of such redundant control mechanisms, low-dimensional coordinative structures among multiple components have been investigated. However, structures extracted from the conventional statistical dimensionality reduction methods do not reflect dynamical properties in principle. Here we regard coordinative structures in biological periodic systems with unknown and redundant dynamics as a nonlinear limit-cycle oscillation, and apply a data-driven operator-theoretic spectral analysis, which obtains dynamical properties of coordinative structures such as frequency and phase from the estimated eigenvalues and eigenfunctions of a composition operator. Using segmental angle series during human walking as an example, we first extracted the coordinative structures based on dynamics; e.g. the speed-independent coordinative structures in the harmonics of gait frequency. Second, we discovered the speed-dependent time-evolving behaviours of the phase by estimating the eigenfunctions via our approach on the conventional low-dimensional structures. We also verified our approach using the double pendulum and walking model simulation data. Our results of locomotion analysis suggest that our approach can be useful to analyse biological periodic phenomena from the perspective of nonlinear dynamical systems.

## Introduction

Living organisms dynamically and flexibly operate a great number of components such as neurons, muscle fibers and skeletal joints. These phenomena can be seemingly regarded as dynamical systems based on some specific rules; however, it is often intractable to find optimal solutions due to a number of combinations of controlled elements and environments. In the field of neuroscience or motor control, for example, this problem has been referred to as Bernstein’s degrees of freedom problem or redundant problem^1^. For this problem, studies in a forward modeling approach have accomplished forward simulations by introducing various bio-inspired systems^2–4^, and by solving parameter optimisation problems^5,6^. However, the governing equation of a real organism behaviour has sometimes been complicated or unclear. Also in other fields, there are many examples of periodic systems with unknown and redundant dynamics, such as population dynamics in ecology and epidemiology and collective motion dynamics in animals and artificial agents. A backward or data-driven approach, which is expected to extract essential information from observed data, would be an effective way to understand them.

One of the popular research subjects of the unknown and redundant dynamic phenomena is human locomotion, which can be performed at various speeds by solving the redundancy problem of many skeletal joints. This is because it seems to be based on specific rules (i.e. can be regarded as a cyclic movement) but the governing equation is not fully understood; thus, from a long time ago, it has attracted attention in many scientific and engineering fields such as neuroscience^7,8^, physics^9,10^, clinical medicine^11,12^, behavioural science^13,14^, robotics^4,6^ and pattern recognition^15^. For the redundant problem, some researchers have discovered synergistic and global structures among controlled components called *coordinative structures* (or low-dimensional spatially coherent structure) in joint motion^8^ and muscle activities^7,16^. Computing such coordinative structure may contribute to understanding the redundant problem in many fields. In the early days, for locomotion, researchers have found that the three-dimensional trajectory of elevation angles of limb segments lies close to a two-dimensional plane^8,17^, and have suggested that this planar law (or low-dimensional structure) may reflect intersegmental coordination. The planar law of intersegmental coordination has been observed at different walking speeds^18^, forward or backward direction^19^ and different levels of body weight unloading^20^. It has also been observed in cats^21^, monkeys and human infants^22^. The idea of the low-dimensional structures was also supported by the successful clinical application^12^ and the accomplishment of walking model simulation^23^. However, the conventional methods to extract coordinative structures have used statistical dimensionality reduction methods with assumptions of independence of sampling such as principal component analysis (PCA). The problem is that the extracted structure does not reflect dynamical properties in principle and still, there has been little theoretical progress in this field. Therefore, an extraction method of the coordinative structures based on dynamical properties from data is needed. In other words, our motivation in this study is to understand the global dynamics behind the data obtained from human locomotion, of which governing equations are not fully known (e.g. neural inputs: for details, see Supplementary Text S5).

As a method to obtain a global modal description of nonlinear dynamical systems, operator-theoretic approaches have attracted attention such as in applied mathematics, physics and machine learning. One of the approaches is based on the composition operator (usually referred to as the Koopman operator^24,25^), which defines the time evolution of observation functions in a function space. The advantage to use the operator-theoretic approach is to lift the analysis of nonlinear dynamical systems to a linear (but infinite-dimensional) regime, which is more amenable to the subsequent analysis. For example, spectral analysis of the Koopman operator can obtain dynamical properties which we define as physical properties regarding time-evolving behaviour such as frequencies with delay/growth rate and coordinative (or coherent) structures corresponding to these properties. Among several estimation methods, one of the most popular algorithms for spectral analysis of the Koopman operator is dynamic mode decomposition (DMD)^26,27^. The benefit of DMD is to extract such a global modal description of a nonlinear dynamical system from data, unlike other unsupervised dimensionality reduction methods such as PCA or singular value decomposition (SVD) for static data. The extracted coordinative structure based on the above dynamical properties via DMD is called *DMD modes*. Among many variants of DMD to overcome the drawbacks of basic DMDs (for details, see Materials and Methods), Hankel DMD^28^ theoretically yields the eigenvalues and eigenfunctions of a Koopman operator (called *Koopman eigenvalues* and *eigenfunctions*) by applying DMD to a Hankel matrix (or delay embedding matrix). The eigenfunction, which provides linearly evolving coordinates in the underlying state space^29^, can describe the phase in (asymptotically) stable dynamical systems such as a classical Van del Pol oscillator model^28,30^. Although there was no research which applied Hankel DMD to biological periodic data without unknown and redundant dynamics, Hankel DMD has the potential to reveal the dynamics in such periodic systems such as human locomotion. Therefore, the first contribution of our approach is to extract dynamical information such as a frequency, phase and its coefficients (i.e. coordinative structures) for the understanding of biological periodic systems with redundant and unknown governing equations (e.g. neural inputs). Furthermore, we propose a new algorithm to compute coordinative structure in the conventional Hankel DMD (for details, see Materials and Methods).

From the perspective of phase reduction, which has been studied in mathematics^31^ and biology^32^, we can also regard the underlying coordinative structure as a dynamical system with a limit cycle and reduce a periodic system to a phase model using our approach. In phase reduction, a limit-cycle oscillator (possibly evolving in high-dimensional space) is approximated to a phase oscillator (evolving on a one-dimensional circle) which is more amenable to mathematical analysis. Phase reduction has been used to analyze such as neural network models^33^ and a circadian gene network model^34^, but there have been fewer direct applications to the actual biological systems with unknown governing equations. Other researchers^35,36^ reviewed the legged locomotion dynamics including a phase reduction model of oscillators, but that of an actual biological (e.g. intersegmental) motion in a data-driven manner was not examined. Therefore, the second contribution of our approach is to obtain a phase reduction model in periodic movements of living organisms with unknown governing equations by a data-driven, operator-theoretic spectral analysis. Compared with the conventional approaches for collective motion dynamics with unknown governing equations (e.g. using a DMD variant^37^), we adopt Hankel DMD, which has strong theoretical support to understand the biological systems as dynamical systems or a phase model. In this study, we confirmed that the theoretical requirements are satisfied for locomotion data from the viewpoint of sufficiently small estimation error (for details, see Supplementary Text S1). Although other locomotion studies obtained phase descriptions using local joint angles^10,38,39^, or a global one based on gait cycles^40^, in this study, we obtain global phase descriptions with various frequencies from locomotion data at various speeds, which provides beneficial information for understanding the global dynamics in a data-driven manner. Furthermore, we examine the difference and similarity between the conventional and our approaches by investigating the extracted dynamical information on the conventional coordinative structures.

The purpose of this research is to clarify the dynamical properties of coordinative structures in periodic data with (partly) unknown and redundant dynamics by a data-driven spectral analysis of nonlinear dynamical systems called Hankel DMD. Our approach can provide conventional dimensionality reduction approaches computing coordinative structure to dynamical information with physical meaning. To this end, we comprehensively validate that our approach can obtain meaningful dynamical properties of coordinative structures in terms of DMD mode, eigenvalues, Koopman eigenfunction with comparisons among various walking speeds and with various existing methods and simulation data. In particular, as a phase reduction method, row-type Hankel DMD described below is useful from the above viewpoints. In the next subsections, we briefly describe basic DMD to understand Hankel DMDs. Thereafter, we present the results of an application of (Hankel) DMDs on actual human locomotion. Additionally, in supplementary materials, we validate our method using simulation data of less redundant system than actual human locomotion: a double pendulum with a small initial condition (has explicit analytical solutions and no redundancy) and a walking model (has explicit governing equations and less redundancy than the actual human walking).

## Background of DMDs

Here, we first briefly review the underlying theory for DMD called Koopman spectral analysis, and then describe the basic and Hankel DMD procedures. In Koopman spectral analysis, we consider a nonlinear dynamical system: ***x***_*t*+1_ = ***f***(***x***_*t*_), where ***x***_*t*_ is the state vector in the state space ${\mathcal M} \subset {{\mathbb{R}}}^{p}$ with time index $t\in {\mathbb{T}}:={{\mathbb{N}}}_{0}$. *The Koopman operator* ${\mathscr{K}}$ is a linear operator acting on a scalar observable function $g: {\mathcal M} \to {\mathbb{C}}$ defined by

1 $${\mathscr{K}}g=g\,\circ \,{\boldsymbol{f}},$$

where $g\,\circ \,{\boldsymbol{f}}$ denotes the composition of *g* with ***f***^24^. We assume that ${\mathscr{K}}$ has only discrete spectra. Then, it generally performs an eigenvalue decomposition: ${\mathscr{K}}{\phi }_{j}({\boldsymbol{x}})={\lambda }_{j}{\phi }_{j}({\boldsymbol{x}})$, where ${\lambda }_{j}\in {\mathbb{C}}$ is the *j*-th (Koopman) eigenvalue and *φ*_*j*_ is the corresponding (Koopman) eigenfunction. We denote the concatenation of scalar functions as ***g***: = [*g*_1_, …, *g*_*d*_]^T^. If each *g*_*i*_ lies within the space spanned by the eigenfunction *φ*_*j*_, we can expand the vector-valued ***g*** in terms of these eigenfunctions as ${\boldsymbol{g}}({\boldsymbol{x}})={\sum }_{j=1}^{\infty }\,{\phi }_{j}({\boldsymbol{x}}){\psi }_{j}$, where ***ψ***_*j*_ is a set of vector coefficients called *the Koopman modes*. Through the iterative applications of ${\mathscr{K}}$, the following equation is obtained:

2 $${\boldsymbol{g}}({{\boldsymbol{x}}}_{t})=({\boldsymbol{g}}\circ \mathop{\underbrace{{\boldsymbol{f}}\circ \cdots \circ {\boldsymbol{f}}}}\limits_{t})({{\boldsymbol{x}}}_{0})=\mathop{\sum }\limits_{j=1}^{\infty }\,{\lambda }_{j}^{t}{\phi }_{j}({{\boldsymbol{x}}}_{0}){\psi }_{j}\mathrm{.}$$

Therefore, *λ*_*j*_ characterises the time evolution of the corresponding Koopman mode ***ψ***_*j*_, i.e. the phase of *λ*_*j*_ determines its frequency and the magnitude determines the growth rate of its dynamics.

**Figure 1:**
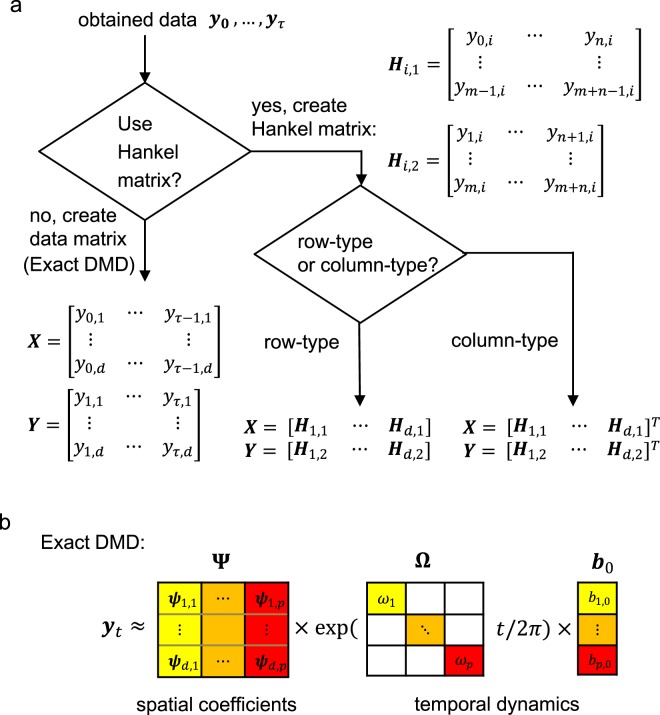
Schematic diagram of DMDs. (**a**) Using obtained data, for exact DMD (a basic DMD), we create data matrices. For Hankel DMD, we first create Hankel matrices, and concatenate the Hankel matrices. We indicate two Hankel DMDs: row-type and column-type. For these three DMDs, the following computation to obtain DMD eigenvalues is the same (regarding companion-matrix DMD and computation of DMD modes for these DMDs, see Background of basic DMDs and Hankel DMDs). (**b**) Diagram of input and output of exact DMD. DMDs decompose obtained data sequences into spatial coefficients and time dynamics. For other DMDs, the diagrams are different: see the main text.

Among several possible methods to compute the above modal decomposition from data, DMD^26,27^ is the most popular algorithm, which estimates an approximation of the decomposition in Eq. (2). Consider a finite-length observation sequence ***y***_0_, ***y***_1_, …, ***y***_*τ*_ ($\in {{\mathbb{C}}}^{d}$), where ***y***: = ***g***(***x***_*t*_). Let ***X*** = [***y***_0_, ***y***_1_, …, ***y***_*τ*−1_] and ***Y*** = [***y***_1_, ***y***_2_, …, ***y***_*τ*_] (without creating Hankel matrix: see Fig. 1a). Then, DMD basically approximates it by calculating the eigendecomposition of matrix ${\boldsymbol{F}}={\boldsymbol{Y}}{{\boldsymbol{X}}}^{\dagger }$, where ${{\boldsymbol{X}}}^{\dagger }$ is the pseudo-inverse of ***X***. The matrix ***F*** may be intractable to analyze directly when the dimension is large. Therefore, in the popular implementation of DMD called exact DMD^41^, a rank-reduced representation $\hat{{\boldsymbol{F}}}$ based on SVD is applied. That is, ***X*** ≈ *U*ΣV^*^ and $\hat{{\boldsymbol{F}}}$ = U^*^FU = U^*^YVΣ^(−1)^, where ^*^ is the conjugate transpose. Thereafter, we perform eigendecomposition of $\hat{{\boldsymbol{F}}}\in {{\mathbb{C}}}^{p\times p}$ to obtain the set of the eigenvalues *λ*_*j*_ and eigenvectors ***w***_*j*_. Then, we estimate the Koopman modes in Eq. (2): ${\psi }_{j}={\lambda }_{j}^{(-1)}{\boldsymbol{YV}}{{\boldsymbol{\sum }}}^{(-1)}{w}_{j}\in {{\mathbb{C}}}^{d\times 1}$ for *j* = 1, …, *p*, which is called *DMD modes*.

In this study, we consider real-valued input matrices ${\boldsymbol{X}},{\boldsymbol{Y}}\in {{\mathbb{R}}}^{d\times \tau }$, whereas DMD can be applied to complex-valued matrices. Even if we use the real-valued input matrices, since $\hat{{\boldsymbol{F}}}$ is usually a non-Hermitian matrix (in a real-valued case, an asymmetric matrix), eigenvalues and eigenvectors (then, DMD modes) are often complex. Therefore, we used the absolute value of ***ψ***_*i*,*j*_, which is an element of ***ψ***_*j*_ for *i* = 1, …, *d*, for representing the spatial weight of the intersegmental coordinative structures. For visualising DMD spectrum, we scaled each mode as previously described^42,43^. Eigenvalue *λ*_*j*_ is transformed into temporal frequency *ω*_*j*_ such that *ω*_*j*_ = *ln*(*λ*_*j*_)/2*π*Δ*t*, where Δ*t* is a time interval of the discrete time system. Time dynamics of *j* th mode, which corresponds to the temporal coordination in the conventional decomposition method using SVD (we call it SVD-based method), is defined as exp(*ω*_*j*_*t*/2*π*)*b*_*j*,0_, where ${b}_{j\mathrm{,0}}={{\boldsymbol{\psi }}}_{j}^{\dagger }{{\boldsymbol{y}}}_{0}$^42^ and ${{\boldsymbol{\psi }}}_{j}^{\dagger }$ is the *j*-th row of the pseudo-inverse of [***ψ***_1_ … ***ψ***_*p*_]. In summary, obtained time series data is decomposed into spatial coefficients and temporal dynamics(Fig. 1b): ${{\boldsymbol{y}}}_{t}\approx {\sum }_{j\mathrm{=1}}^{p}\,{{\boldsymbol{\psi }}}_{j}{\rm{e}}{\rm{x}}{\rm{p}}({\omega }_{j}t/2\pi ){b}_{j\mathrm{,0}}={\boldsymbol{\Psi }}{\rm{\exp }}({\boldsymbol{\Omega }}t/2\pi ){{\boldsymbol{b}}}_{0}$, where **Ψ** is a DMD mode matrix, **Ω** are diagonal matrix consisting of *ω*_*j*_ and ***b***_0_ is a vector consisting of *b*_*j*,0_.

Theoretically, each observable function *g*_*i*_ should lie within the space spanned by the Koopman eigenfunction *φ*_*j*_, i.e. the data should be rich enough to approximate the eigenfunctions. However, in basic DMD algorithms naively using the obtained data such as both exact and companion-matrix DMD (see Materials and Methods), the above assumption is not satisfied such as when the data dimension is too small to approximate the eigenfunctions. Thus, there are several algorithmic variants (for detail, see Materials and Methods), including Hankel DMDs.

Hankel DMD^28^ is a variant of DMD applied to delay embedding matrix, which can theoretically yield Koopman eigenvalues and eigenfunctions. Conventional Hankel DMD duplicates the original data by delay embedding (Fig. 1a), i.e. uses the Hankel matrices of data in the forms of

3 $${{\boldsymbol{H}}}_{i,1}=[\begin{array}{cccc}{y}_{0,i} & {y}_{1,i} & \ldots  & {y}_{n,i}\\ {y}_{1,i} & {y}_{2,i} & \ldots  & {y}_{n+1,i}\\ \vdots  & \vdots  & \ddots  & \vdots \\ {y}_{m-1,i} & {y}_{m,i} & \ldots  & {y}_{m+n-1,i}\end{array}],\,{{\boldsymbol{H}}}_{i,2}=[\begin{array}{cccc}{y}_{1,i} & {y}_{2,i} & \ldots  & {y}_{n+1,i}\\ {y}_{2,i} & {y}_{3,i} & \ldots  & {y}_{n+2,i}\\ \vdots  & \vdots  & \ddots  & \vdots \\ {y}_{m,i} & {y}_{m+1,i} & \ldots  & {y}_{m+n,i}\end{array}],$$

for *i* = 1, 2, …, *d*, where *y*_*t*,*i*_ is an element of vector ${{\boldsymbol{y}}}_{t}\in {{\mathbb{C}}}^{d}$ for *t* = 0, …, *τ*. Using these Hankel matrices, we compute the modified Hankel DMDs according to the aim of this study. In summary, we investigated two types of Hankel DMDs (Fig. 1: row-type and column-type Hankel DMDs. Row type Hankel DMD used input concatenated matrices ***X***_*H*1_ = [***H***_1,1_, ***H***_2,1_, …, ***H***_*d*,1_] and ***Y***_*H*1_ = [***H***_1,2_, ***H***_2,2_, …, ***H***_*d*,2_]. We compute this row-type Hankel DMD for estimating Koopman eigenfunctions corresponding to one-dimensional dynamics. In a more straightforward way to compute the coordinative structure, column-type one used the matrices ***X***_*H*2_ = [***H***_1,1_, ***H***_2,1_, …, ***H***_*d*,1_]^T^ and ***Y***_*H*2_ = [***H***_1,2_, ***H***_2,2_, …, ***H***_*d*,2_]^T^. However, it is not straightforward for column-type Hankel DMD to estimate a one-dimensional eigenfunction for each dynamics from the viewpoint of phase model.

For the guidance on how Hankel DMDs should be used, we explain the detailed procedures including the preprocessing in Materials and Methods. The actual procedure can be confirmed using the Matlab code available at https://github.com/keisuke198619/HankelDMD. For the selection of the parameters of Hankel DMDs such as delay embedding dimension and the truncation dimension (i.e. number of DMD modes), we present the details including the evaluation with the various parameters in Supplementary Texts S1 and S2. Hankel DMDs decompose the augmented data into DMD modes, eigenvalues and eigenfunctions. We present and evaluate the results in this order. Regarding the redundancy of the system to be analyzed, we also investigated double pendulum and walking model simulation data (less redundant than the actual human locomotion in this order).

## Results

### Decomposition of segmental angles

**Figure 2:**
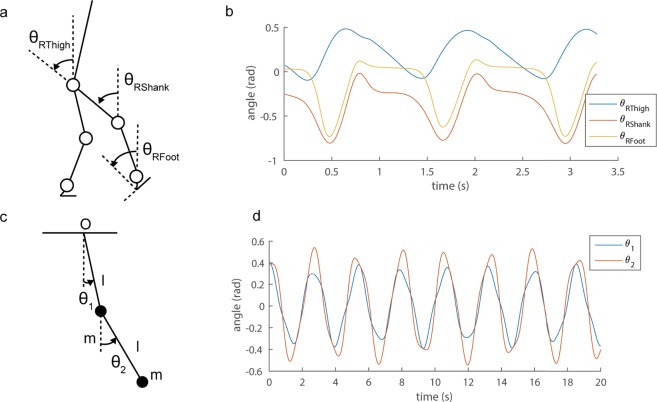
Angle definitions and examples of angle time-series. Definition of angles of human body (**a**) and double pendulum (**c**) are shown. Examples of angle time-series for (**b**) human locomotion^61^ and (**d**) double pendulum^62^ are indicated.

**Figure 3:**
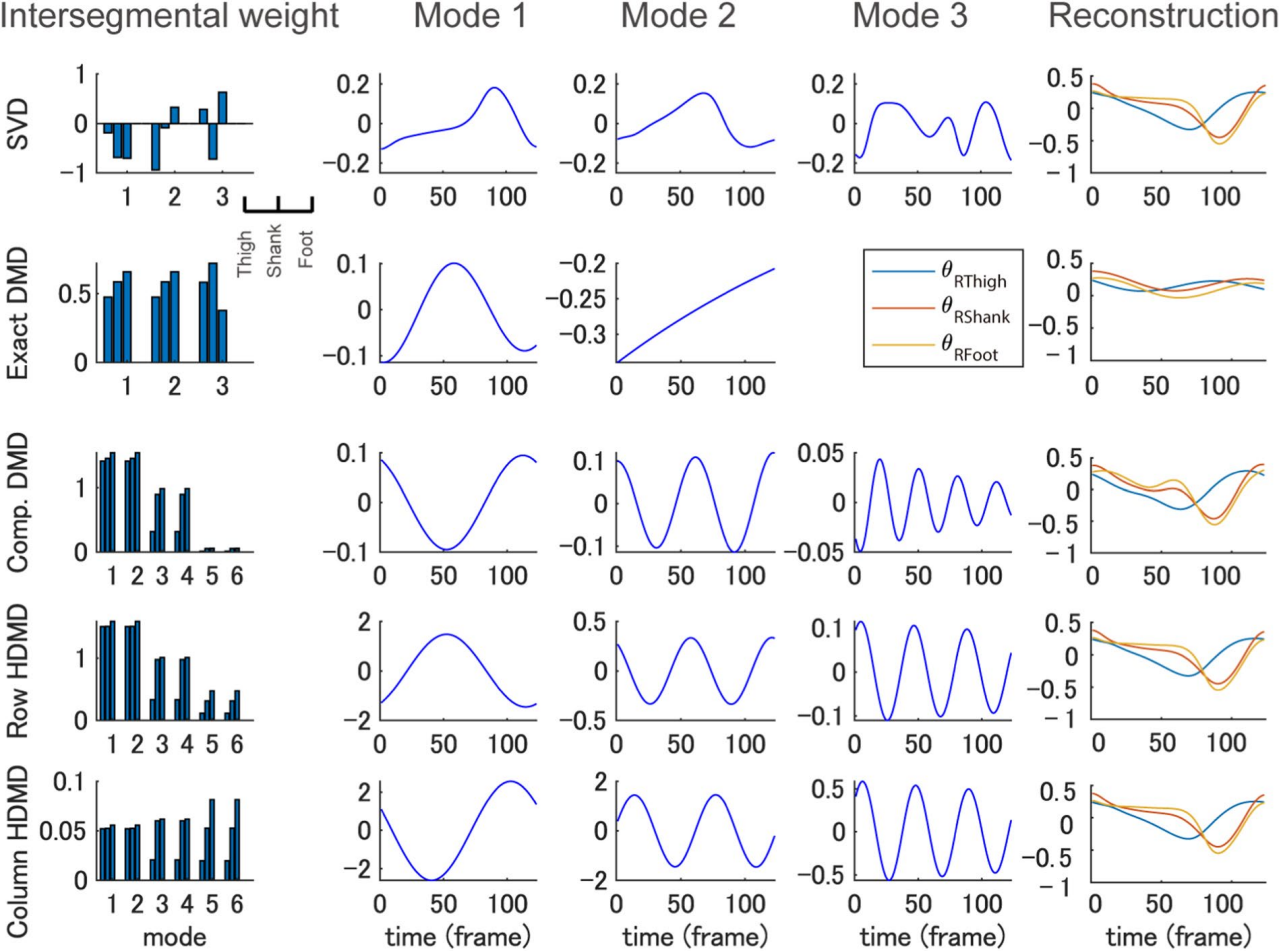
Representative examples of five decomposition methods. Examples of decomposition results into intersegmental weights (or DMD modes) in column 1 and time dynamics in the center three columns by conventional SVD-based method (row 1), exact DMD (row 2), companion-matrix DMD (row 3), row-type Hankel DMD (row 4) and column-type Hankel DMD (row 5) during a 2.0 km/h walk are shown. The rightmost column shows the reconstructed angle time-series data (the input data was computed by subtracting the temporal average values from the original data). SVD-based method shows similar two principal components in the previous study^44^. Exact DMD shows that only two conjugate modes and one mode because of the data dimension *d* = 3. In companion-matrix DMD and the two Hankel DMDs, we show only three pairs of conjugate modes that show the highest VAF. Note that all DMDs compute complex-valued DMD modes and time dynamics, but here we only illustrate the absolute value and real part of them, respectively. VAF and reconstruction error of the dominant modes were higher and lower for SVD-based method, column-type Hankel DMD, row-type Hankel DMD, companion-matrix DMD and exact DMD in this order. Sampling frequency was 100 Hz.

In this subsection, we show examples of the applications of our approach, and evaluated our methods such as using reconstruction error. First, we show an example of segmental angles in human locomotion in Fig. 2a,b, and show representative examples of intersegmental coordination by five decomposition methods in Fig. 3: conventional SVD-based method, basic two DMDs and two Hankel DMDs for each row. In the analysis, we used three elevation angles of the right thigh, shank and foot in one cycle during 2.0 km/h walk for a participant (*T* = 126 [frame]), according to the previous study^8^ (for other experimental setups, see Materials and Methods).

To understand the difference between the conventional and our approaches, we first show the results of conventional SVD-based method (for the detailed procedure, see Supplementary Text S4). SVD-based method in the first row of Fig. 3 shows similar two principal components in the previous study^44^. For evaluation, we used variability accounted for (VAF) and reconstruction error which are commonly used in non-orthogonal dimensionality reduction methods (for details, see Materials and Methods). VAF was also similar to the previous work in which on average the first two principal components accounted for more than 99% of the data (the mean reconstruction error for all walking speeds in three components is 0.0243 ± 0.0043 rad). However, SVD-based method cannot obtain the dynamical information that can be obtained by DMDs.

Next, we show that Hankel DMD can extract the dynamical information from data. We compute Hankel DMDs for two different computational procedures (row- and column-type: see Fig. 1). For understanding, we first show the results of basic DMDs (exact and companion-matrix DMDs). Exact DMD in row 2 of Fig. 3 using the same data matrix in SVD-based method shows that only two conjugate modes and one mode because of the constraint of data dimension *d* = 3. Note that all DMDs compute complex-valued DMD modes and time dynamics, but here we only show the absolute value and real part of them, respectively. Algorithmically, companion-matrix DMD row 3 of Fig. 3 does not have the constraints; however, since the data dimension is too small to approximate the eigenfunctions, the two basic DMDs did not satisfy the theoretical requirements (based on the reconstruction error analysis below). Hankel DMDs in rows 4 to 5 of Fig. 3 satisfied this requirements using delay-embedding matrix (i.e. by augmenting the data) based on the better reconstruction than the basic DMDs as explained below.

Here, for clarity, we show only three pairs of conjugate modes which show the highest VAF in this order. Although all four DMDs extracted a similar pair of temporal dynamics with approximately one cycle (in column 2 of Fig. 3), companion-matrix DMD and the two Hankel DMDs additionally extracted those with approximately two and three (or four) cycles.

Regarding VAF and reconstruction error of the dominant modes, column-type Hankel DMD shows the best performance among the DMDs in the rightmost column of Fig. 3. Note that we performed the error analysis for the evaluation within DMD approaches and we cannot fairly compare SVD-based method and DMD approaches for the analysis (for details, see Discussion). On average among all walking speeds and participants, VAF was −9.76 ± 6.35%, 26.70 ± 16.94%, 76.13 ± 0.59% and 76.18 ± 0.48%, and reconstruction error was 0.3485 ± 0.0597, 0.2891 ± 0.0716, 0.0393 ± 0.068 and 0.0365 ± 0.074 rad for exact (a pair of the modes), companion-matrix, row-type and column-type Hankel DMDs (three pairs), respectively. Statistical analysis indicates significant main effects of methods for both error (*χ*_4_^2^ = 459.63, *p* = 2.210^−16^, *W* = 0.91) and VAF (*χ*_4_^2^ = 478.9, *p* = 2.210^−16^, *W* = 0.95). Subsequent multicomparison shows significant differences among all combinations of the methods for error and VAF (*p* < 1.410^−14^, |*r*| > 0.50), except for the VAF between row- and colomn-type Hankel DMDs (*p* = 0.51, *r* = −0.04). Data for all walking speeds are shown in Supplementary Figs S8 and S9.

As a dynamical system to obey explicit governing equations, the results of the double pendulum (Fig. 2c,d) and walking model simulation data (the models are described in Supplementary Text S5) are shown in Supplementary Figs S3 and S4, respectively. Note that although in reconstruction error SVD obviously outperformed any DMD method, DMD methods can extract dynamical information that SVD cannot obtain, as shown in the below subsection.

### Hankel DMD modes

**Figure 4:**
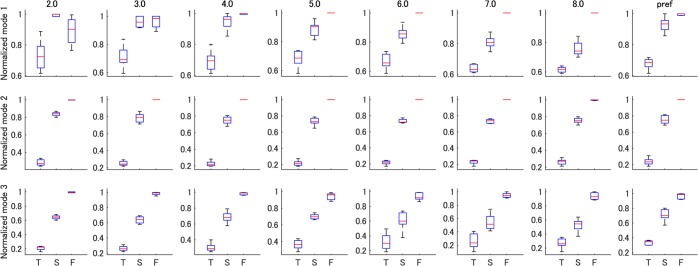
Boxplot of row-type Hankel DMD Modes at various speeds. Normalised row-type Hankel DMD Mode 1 to 3 averaged within walking participants for eight velocities are shown. Pref. indicates preferred walking speed. T, S and F indicate thigh, shank and foot angles, respectively. T, S and F indicate thigh, shank and foot angles, respectively. The DMD modes were similar among four velocities in terms of the larger weight of foot angle than that of thigh and shank angles, except for the mode 1 at low walking speeds.

Next, we interpreted the extracted Hankel DMD modes (or coordinative structures) among the intersegmental angles. Especially, we investigated whether a speed-independent or -dependent coordinative structure exists in locomotion angle data at various walking speeds. In column 1 of Fig. 3, those were similar among three DMDs in larger weight of foot angle than those of thigh and shank angles. As shown in Fig. 4, this tendency was consistent among various walking speeds in row-type Hankel DMD (the results of column-type Hankel DMD were similar, which are shown in Supplementary Fig. S10. For quantitative comparison, we normalised the absolute values of modes because the amplitude of modes depends on that of the time dynamics. Statistical analysis indicates significant main effects of modes for all time dynamics (*χ*_2_^2^ > 208.32, *p* < 2.210^−16^, *W* > 0.82). Subsequent multicomparison shows significant differences among all combinations of modes for all time dynamics (*p* < 0.910^−12^, |*r*| > 0.44), as shown in Fig. 4. The results indicate that foot angles in walking robustly contributed to the global dynamics at harmonic frequencies more than thigh and shank angles, except for the mode 1 at low walking speeds.

### DMD (Koopman) eigenvalues

**Figure 5:**
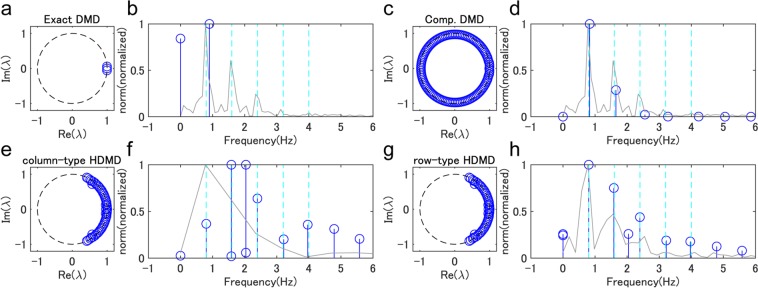
Representative examples of eigenvalues in four decomposition methods. Results of DMD eigenvalues (blue circles) in a complex plane (a,c,e, and g) and DMD and Fourier spectrum (b,d,f and h) for exact DMD (**a** and **b**), companion-matrix DMD (**c** and **d**), column- (**e** and **f**) and row-type Hankel DMD (g and h) are shown. We used the same example of Fig. 3. DMD and Fourier spectra are shown as blue and gray lines, respectively. Harmonic frequencies of the gait frequency are also shown as light blue dashed line.

Next, we show that Hankel DMDs can extract the dynamical properties regarding temporal frequencies behind segmental angles during locomotion. In particular, we investigated the speed-dependency of the dynamical properties and the difference between various DMDs and Fourier transformation. First, we explain it using representative distributions of eigenvalues and temporal frequency spectra computed by exact DMD, companion-matrix DMD, and column- and row-type Hankel DMDs in Fig. 5 (we used the same example of Fig. 3). For all DMDs, all eigenvalues were almost on the unit circle; however, the distributions were different. Both types of Hankel DMD in Fig. 5e,g with the dimension of the truncation *p* = 50 (the number of eigenvalues) show a similar distribution (this truncation was worked as a low-pass filter in the temporal frequency domain; the reason is described in Supplementary Text S1) whereas the eigenvalues of companion-matrix DMD in Fig. 5c (*p* = *T* = 126 without truncation) uniformly occupied on the unit circle and those of exact DMD in Fig. 5a (*p* = *d* = 3 without truncation) were near the real axis. Next, in the temporal frequency domain, we show DMD and Fourier spectra averaging among all data dimensions of the input matrix. Although exact DMD in Fig. 5b extracted low-frequency component among Fourier spectra, other DMDs obtained five or more harmonic frequencies (shown as light blue dashed line) of the gait frequency. In particular, row-type Hankel DMD in Fig. 5h can extract similar spectra to the averaged Fourier spectra, whereas the higher-frequency norms of companion-matrix DMD in Fig. 5d decayed in higher frequencies and the spectra of column-type Hankel DMD in Fig. 5f were relatively different from the averaged Fourier spectra.

**Figure 6:**
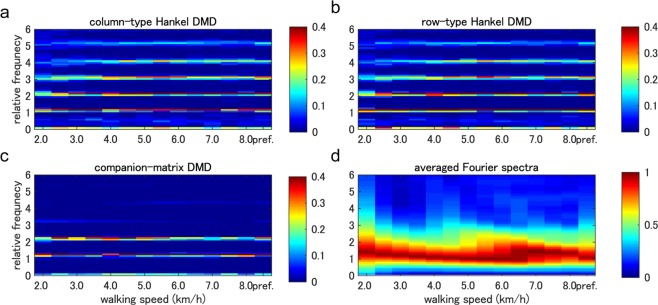
Extracted frequencies in four decomposition methods. Temporal frequency spectra averaged among all participants in relative frequency domain to the gait frequency for (**a**) column-type and (**b**) row-type Hankel DMDs, (**c**) companion-matrix DMD and (**d**) Fourier transformation for all walking speeds are shown. Pref. indicates preferred walking speed. The spectrum of each sequence was normalised so that the maximal norm is 1. For all walking speeds, column- and row-type Hankel DMDs visually obtained five harmonic frequencies. However, companion-matrix DMD visually shows only two harmonic frequencies. Averaged Fourier spectra were seemingly smoothed around the gait frequency.

Next, we visually and quantitatively evaluated the extraction of the harmonic frequencies in Fig. 6. We show the averaged spectrum among all participants of the normalised spectrum of each sequence so that the maximal norm is 1. Since the gait frequencies during walking with the same speed were slightly different within and among participants, we normalised the frequency by dividing by the gait frequency. For all walking speeds, column- and row-type Hankel DMDs in Fig. 6a,b visually obtained five harmonic frequencies (we denote the estimated five frequencies as *ω*_1_, …, *ω*_5_). However, companion-matrix DMD in Fig. 6c shows (visually) only two harmonic frequencies. Note that we did not show the result of exact DMD because of the fewer extracted dynamics in Fig. 5. Then, we quantitatively evaluated the difference between the obtained frequencies and the harmonic frequencies of the gait frequency after the normalisation by dividing by the harmonic frequencies. These differences in companion-matrix DMD (neglecting missing values) and column-type and row-type Hankel DMDs were 0.2655 ± 0.0427, 0.0213 ± 0.0100 and 0.0213 ± 0.0100 rad/s, respectively. We did not perform statistical analysis because the result of companion-matrix DMD had many missing values (the results of column- and row-type Hankel DMDs were numerically almost the same). Since averaged Fourier spectra in Fig. 6d were seemingly smoothed around the gait frequency (continuous spectra), we did not quantitatively evaluate the averaged Fourier spectra. Also, the results of the double pendulum simulation data shown in Supplementary Fig. S5 indicate that both types of Hankel DMDs extracted the two eigenfrequencies computed by the analytical solution. Moreover, in the walking simulation data shown in Supplementary Fig. S6, we obtained similar results to Fig. 6.

### Koopman eigenfunctions

**Figure 7:**
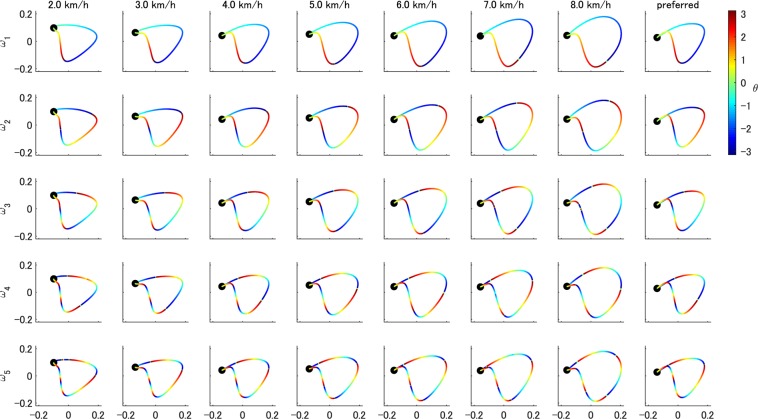
Phases of the Koopman eigenfunctions estimated by row-type Hankel DMD on the conventional coordinative structures. Phases computed as the argument of the Koopman eigenfunctions estimated by row-type Hankel DMD are shown for each walking speed and five harmonic frequencies of a participant. Horizontal and vertical axes are principal axes extracted by SVD-based method. For clarity, we aligned the initial phases to near-zero values by the time-shift for all eigenfunctions. All trajectories rotate clock-wise from the black dots. Although the shapes were similar among various velocities, the changes in the phase with walking speed (or gait cycles) were inhomogeneous. For example, the phases visually advanced with the increase of the walking speed, such as in the boundary between blue and red at the bottom in the plots of *ω*_1_ (but the phase did not seemingly change in the boundary between red and yellow).

Next, in Fig. 7, we visualised the speed-dependent time-evolving behaviour of the phase of Koopman eigenfunction (in color, by row-type Hankel DMD) on the trajectory obtained by SVD-based method (i.e. in principal coordinates) to understand the dynamics on the conventional low-dimensional structure. This is one of the advantage of row-type Hankel DMD (note that column-type Hankel DMD cannot extract the eigenfunction corresponding to the one-dimensional dynamics). Specifically, we investigated the speed-dependency of the phase behavior. The Koopman eigenfunctions ${\tilde{{\boldsymbol{\varphi }}}}_{l}$ computed via row-type Hankel DMD are equivalent to the columns of the Hankel DMD modes corresponding to the focused dynamics (${\tilde{{\boldsymbol{\varphi }}}}_{l}$ s for *l* = 1, ..., 5 correspond to *ω*_1_, ..., *ω*_5_). We computed the argument of the complex-valued Koopman eigenfunction ${\boldsymbol{\theta }}{\boldsymbol{^{\prime} }}=\angle {\tilde{{\boldsymbol{\varphi }}}}_{l}$ as previously shown^28^. For clarity, we aligned the initial phases to near-zero values by the time-shift for all the eigenfunctions. Thus, all trajectories in Fig. 7 for a participant rotate clock-wise from the black dots.

Results show that, although the shapes in Fig. 7 were similar among various velocities, the time-evolving behaviours of the phase with walking speed (or gait cycles) were heterogeneous. For example, the phases of ${\tilde{{\boldsymbol{\varphi }}}}_{l}$ visually advanced with the increase of the walking speed, such as in the boundary between blue and red at the bottom in the plots of *ω*_1_ (but the phase did not seemingly change in the boundary between red and yellow). One of the possible reasons may be the decrease in the proportion of stance phase (the earlier phase in this case) and the increase in the proportion of swing phase (the later phase) as the walking speed increases^45^. This would be generated from the faster flexion/extension of the three joints as previously illustrated^46,47^. We also discuss this point further below.

Next, we quantitatively evaluated our results by comparing them to the eigenfunction obtained from another method called Fourier (or harmonic) average^30^. Fourier average requires prior knowledge of the frequency (in this case, the accurate harmonic frequencies of the gait frequency) and the Koopman eigenfunction **ϕ**_*l*_ computed via Fourier average was the ideal oscillator without the growth/decay dynamics. We adjusted the initial phases of the Koopman eigenfunction ${\tilde{{\boldsymbol{\varphi }}}}_{l}$ to those of **ϕ**_*l*_. The mean differences of two methods among all the time and sequences were small: for *ω*_1_, …, *ω*_5_ among all speeds and participants, they were 0.0281 ± 0.0117, 0.0120 ± 0.0059, 0.0068 ± 0.0033, 0.0021 ± 0.0015 and 0.0025 ± 0.0013 rad/s, respectively. We did not perform statistical analysis because the difference is substantially (not statistically) near zero. We also visualised the time-evolving behaviour of the phase for the double pendulum and walking model simulation data in Supplementary Fig. S7.

## Discussion

The objective of this study was to reveal dynamical properties of coordinative structures in biological periodic systems with unknown and redundant dynamics by a data-driven spectral analysis of dynamical systems (i.e. Hankel DMD). As a phase reduction method, row-type Hankel DMD is theoretically the only method. To compute only DMD modes and eigenvalues, we show that we can also use column-type Hankel DMD based on the results of DMD modes, eigenvalues and reconstruction error. By comparing with the model simulation data, we obtained similar results of Hankel DMD between the actual human walking and walking model simulation data regardless of the redundancy of the system (7-link with unknown inputs and 5-link with explicit inputs, respectively). In this section, for each paragraph, we discuss the results of the extracted harmonics of gait frequencies, the comparison with the conventional coordinative structures, the extracted properties as dynamical systems and the future perspectives. We then follow up with conclusions.

First, we extracted the harmonics of the gait frequency for various walking speeds in Fig. 6. Especially, both column- and row-type Hankel DMDs extracted the distinct first to fifth harmonics compared with other DMD methods. In general, a periodic function that satisfies Dirichlet’s conditions can be expanded in the harmonic trigonometric functions with a constant offset. Regarding (approximately) linear systems, for example, the double pendulum with a small initial condition in Supplementary Fig. S5, indicates only obvious frequency spectra. However, for nonlinear periodic systems, the strength of the spectra of the harmonics is not obvious, whereas the existence of the harmonics is obvious. Although the harmonics during gaits has been previously observed such as in ground reaction forces^48^, there is a possibility of measurement noise to generate the harmonics of the gait frequency^49^. Then, we compared the results of walking model simulation without the measurement noise, and confirmed the similar harmonics in Supplementary Fig. S6, suggesting that the harmonics would be generated from the interactions of multi-link model and environments (e.g. ground and gravity) rather than the measurement noise. For such a redundant dynamical system, the equations of motion of the link model (described in Supplementary Text S5) become complicated as the model approximates a human. Our approach to extract the dynamic properties of the coordinative structures (frequencies, phases and its coefficients) provides conventional dimensionality reduction approaches to dynamical information with physical meaning; e.g. foot angles in walking robustly contributed to the global dynamics at harmonic frequencies more than thigh and shank angles as shown in Fig. 4. Thus, it would be beneficial for the understanding of the underlying dynamics.

Compared with the conventional methods to compute coordinative structures, Hankel DMDs extracted the structures with dynamical properties behind the data. The conventional SVD-based method, which projects data on a low-dimensional subspace with orthogonal basis fitted by the data, showed better performance in terms of reconstruction error than DMDs but did not extract dynamic properties. These two approaches have different strengths and cannot be fairly compared. Instead, we can comprehensively understand the coordinative structures extracted by the two approaches. Then, we here visualised the relation between coordinative structures by SVD-based method (Supplementary Fig. S11a,b) and column-type Hankel DMD (Supplementary Fig. S11c–g). The variance along the third axis (PC3 in Supplementary Fig. S11b) in the space spanned by three principal directions using SVD indicates the deviation from the existing two-dimensional plane^8,17^. Also in the harmonic frequencies obtained by column-type Hankel DMD in Supplementary Fig. S11c–g, the deviation in the PC3 axis was smaller (on average among all harmonic frequencies, participants and speeds, the maximal absolute deviation was 0.0159 ± 0.0101) than that of SVD (0.1469 ± 0.0251). These results indicate that the reconstructed dynamics from Hankel DMD with the harmonic frequencies also lie in the conventional low-dimensional subspace. However, it should be noted that a similar spatial structure on the conventional two-dimensional plane among various walking speeds did not mean the similar phase of the limit cycle, as shown in Fig. 7. In other words, the conventional SVD-based method^23,50^ often normalised the time series to obtain consistent results, but this loses information on time or phase of the limit cycle. In general, at high harmonic frequencies, normalisation of time-series seems to be delayed when walking speed increases as shown in Supplementary Fig. S11 (but actually advanced because of the higher gait frequency or shorter gait cycle as shown in Supplementary Fig. S12). Although the best computation of the phase depends on the purpose, our approach using operator-theoretic spectral analysis can be useful because it can define the phase in terms of the underlying nonlinear dynamical systems.

To investigate the dynamical properties of the underlying dynamical systems such as a limit cycle, our approach has advantages because it can estimate Koopman eigenvalues (frequency or decay/growth rate), eigenfunctions (projection from data space to latent state space) and DMD modes (spatial coefficients). For understanding biological redundant systems as dynamical systems, it should be noted that the governing equations including neuro-musculo-skeletal dynamics are often partly unknown, which would be different from most of the known physical systems. In this case, as discussed above, a data-driven approach such as used in this study would be effective. Other data-driven approaches may also be effective such as curve fitting using the dictionary of the basis functions^51^ and actually, researchers have applied a sinusoidal curve fitting to the intersegmental coordination during locomotion^52^. Meanwhile, our approach is the data-driven operator-theoretic spectral analysis of nonlinear dynamical systems without a prior explicit knowledge. We adopted this approach with the assumption that there are underlying dynamical systems or limit cycles behind the segmental angles during locomotion. In limit cycles, phase description has been developed for locomotion such as using local joint angles^10,38,39^ or right heel-contact cycle as global description^40^. Compared with these studies, we extracted global phases at the gait frequency and at additional harmonic frequencies in a data-driven manner. However, although we can interpret that the double harmonic frequency may relate to a one-step movement (because one gait cycle includes two steps) and higher harmonic frequencies may relate to the impact and/or absorption of the foot contact, we currently did not provide strict mechanical interpretations.

Further studies would be needed at least from four perspectives. One is the application to a control problem using forward simulations. The global entrainment among the neuro-musculo-skeletal system and the environment during locomotion should be further investigated by neuro-musculo-skeletal simulations with neural inputs such as central pattern generator^3^ and reflex action^6^ by feedback from environments. These will contribute to the understanding of the above mechanical interpretation of the harmonic frequencies. The second is related to our assumption that the walking dynamics is on a limit cycle. Actually, walking is sometimes not an ideal cyclic motion; thus we need a method adapting to rhythm shift and perturbation (observed as a phase reset^38,40^ and a phase locking^39^). Our approach can theoretically describe an asymptotic phase in asymptotically stable dynamical systems^28,30^; therefore, it will be possible to apply it to the perturbed or more complicated locomotion^53–55^. The third is the variation within/among participants. Although this study basically investigated the difference among walking speeds, this dynamics is directly related to the gait (and harmonic) frequency. Comparison with other types of locomotion, or other specific participant groups will contribute to the understanding of the redundant and unknown human motor systems as dynamical systems. The last is related to the limitation of this approach. For the dynamical systems with unknown governing equations, we cannot directly evaluate whether our approach can extract true dynamical properties. Thus, a general quantitative validation method for the unknown dynamics is further required.

In this paper, we applied data-driven and operator-theoretic spectral analysis to segmental angles during human locomotion, which can extract coordinative structures based on dynamic properties and obtain a phase reduction model. We adopted Hankel DMD, which theoretically yields the eigenvalues and eigenfunctions of composition (Koopman) operator by augmenting finite data. First, we extracted the coordinative structures based on dynamics; e.g. the speed-independent coordinative structures in the harmonics of gait frequency. Second, we discovered the speed-dependent time-evolving behaviours of the phase (by estimating the eigenfunctions via row-type Hankel DMD) on the conventional low-dimensional coordinative structure. We also verified our approach using the double pendulum and walking model simulation data. Our results of locomotion analysis suggest that our approach can be useful to analyse biological periodic phenomena from the perspective of nonlinear dynamical systems.

## Materials and Methods

### Participants

Ten healthy men (age: 23.3 ± 0.9; height: 171.1 ± 3.44 cm; weight: 64.1 ± 0.63 kg) participated in this study. The participants provided written informed consent to participate in the study after receiving a detailed explanation of the purpose, potential benefits and risks associated with participation. The experimental procedures were conducted in accordance with the Declaration of Helsinki and were approved by the Local Ethics Committee of the Graduate School of Human and Environmental Studies, Kyoto University (Approval number 26-H-22). This data was the same as used in the previous study^46^. The number of participants was determined by previous studies of motion analysis in locomotion^44,50^. We did not use any other required assumptions. We eliminated data of a participant from the analysis because of a lot of missing values in motion capture data.

### Experimental setup and data preprocessing

Participants walked on a treadmill (Adventure 3 PLUS, Horizon, Johnson Health Tech Japan Co., Tokyo, Japan) at 14 different controlled speeds (from 2.0 to 8.0 km/h with 0.5 km/h interval and a preferred walking speed of 4.3 ± 0.63 km/h) that were administered in a random order over the span of 50 gait cycles. To determine the preferred walking speed for each individual participant, we modulated the treadmill speed without showing the walking speed to the participants. The preferred walking speeds were determined at the moment when the participants felt comfortable. We divided all data into validation and test datasets including both 10 sequences for each participant and walking speed (each sequence includes a gait cycle and additional delay embedding, as explained below). We used the validation dataset in the following procedure of determining the parameters of Hankel DMD. Thereafter, we performed all of the remaining analysis using the test dataset.

Kinematic data were recorded using a 3D optical motion capture system with 12 cameras operating at 100 Hz (Optitrak, Northern Digital Inc., Waterloo, Ontario, Canada). This system captured three-dimensional coordinates of reflective markers that were attached to the anatomical landmarks on the participants. The reflective markers that were attached to the participants were positioned at the top, right and left sides of their heads, as well as on the acromions, elbows, wrists, anterior superior iliacspine, posterior superior iliac spine, greater trochanters, medial and lateral epicondyles, medial and lateral malleolus, heels and toes. The measured reflective marker data were low-pass filtered at 8 Hz, which contains the frequency band in the following analyses. We defined one gait cycle as the time between the initial right heel contact and the next right heel contact. We primarily used one gait cycle for the analysis (for details, see Hankel DMD subsection). Similarly, we defined gait frequency as the reciprocal number of a gait cycle. We defined six elevation angles (Fig. 2a,b: right and left feet, shanks and thighs). Our selection of elevation angles was based on the assumption that changes in elevation angles are more stereotypical than relative angles^8,44^. To prepare to extract coordinative structures, we computed mean postures by averaging the elevation angles over a gait cycle and subtracting them from elevation angles for each segment angle^23,50^. For the subsequent analysis, we primarily used three angles (right feet, shank and thigh) to compare the conventional coordinative structures^8,44^, but when we simulated the following five-link biped model, we used four angles (right and left shanks and thighs).

### Reconstruction error and VAF

To evaluate the dimension of the reduction or determine the number of intersegmental coordination, researchers used the cumulative contribution ratio using eigenvalues^23,50^ because of the orthogonal decomposition. However, in this study, to compare with non-orthogonal decomposition methods such as DMDs, we adopted VAF which is commonly used in non-orthogonal dimensionality reduction such as non-negative matrix factorisation in muscle activity^46^. In DMDs, VAF is defined as the square error of the reconstructed data ${\hat{{\boldsymbol{\Theta }}}}_{p}$ (*p* indicated the reconstructed data using *p* modes) and the original data **Θ** such that ${{\rm{VAF}}}_{p}=1-({\Vert {\boldsymbol{\Theta }}-{\hat{{\boldsymbol{\Theta }}}}_{p}\Vert }_{F}^{2})/{\Vert {\boldsymbol{\Theta }}\Vert }_{F}^{2}$, where $\,{\Vert \cdot \Vert }_{F}$ is the Frobenius norm. Moreover, in DMDs, since the augmented data dimension is variable in Hankel DMD, the absolute reconstruction error is defined as $(1/d\tau ){\sum }_{t=0}^{\tau -1}\,{\sum }_{i=1}^{d}\,\Vert {\theta }_{i,t}-{\hat{\theta }}_{i,t}\Vert$, where *θ*_*i*,*t*_ is a scalar element of **Θ**, and ${\hat{\theta }}_{i,t}={\sum }_{j=1}^{p}\,{\psi }_{i,j}{\lambda }_{j}^{t}{b}_{j,0}$ at dimension *i* and time *t*.

### Variants of DMDs

Here we need to explain several variants of DMDs to indicate the necessity of Hankel DMDs. Algorithmically, the number of DMD eigenvalues *p* in exact DMD is limited to the data dimension *d* if $d\ll \tau$ such as in this study. This property in exact DMD suffers from the problem when we use some biological data including more dynamic modes than the data dimension such as multi-link motion in this study. On the other hand, another popular DMD implementation called companion-matrix DMD^26,28^, can solve the problem (*p* is limited to the time length *τ* in this case). Then, we also examined companion-matrix DMD (and exact DMD) to compare with our approach (Hankel DMD below). Companion-matrix DMD^26,28^, is a more straightforward formulation than exact DMD, but sometimes numerically unstable (the procedure is described in Supplementary Text S3).

There are several algorithmic variants of DMDs to overcome the problem of the original DMD such as the use of a dictionary of basis functions^56^ and a formulation in a reproducing kernel Hilbert space^57^. These methods work well when appropriate basis functions or kernels are prepared; however, it is not always possible to prepare such functions if we have no prior knowledge of the underlying dynamics. Another variant has been recently developed using nonlinear transformation in a neural network framework^58^, which does not need prior knowledge about the observable function. However, in this study, we assume that the dynamical system behind the angle series data during walking is a limit cycle, which is a relatively tractable class in dynamical systems. Thus, we adopt a more straightforward approach without nonlinear transformation according to the previous works including a limit cycle^28^ or unknown dynamics^43,59,60^. Hankel DMD^28^ is also a variant of DMD applied to delay embedding matrix, which can theoretically yield Koopman eigenvalues and eigenfunctions. The Koopman eigenfunction provides linearly evolving coordinates in the underlying state space and can describe the phase in (asymptotically) stable dynamical systems^28,30^. Therefore, we adopted Hankel DMD, as described in the next subsection.

### Hankel DMDs

Here, we introduce conventional Hankel DMD and describe the new formulation to compute Hankel DMD modes according to the aim of this study. Hankel DMD^28^ is also a variant of DMD applied to delay embedding matrix, which can theoretically yield Koopman eigenvalues and eigenfunctions. Conventional Hankel DMD duplicates the original data by delay embedding (Fig. 1a), i.e. uses the Hankel matrices of data in Eq. (3). Although in the previous work^28^ the scaling factors were computed for normalising the norms of the observables, the segmental angles in this study were not extremely large or small among the observables; thus we did not use the scaling factors. Next, we form the concatenated matrices: ***X***_*H*1_ = [***H***_1,1_, ***H***_2,1_, …, ***H***_*d*,1_] and ***Y***_*H*1_ = [***H***_1,2_, ***H***_2,2_, …, ***H***_*d*,2_]. Then, we compute the truncated SVD: ***X***_*H*1_ ≈ ***U***_*H*_**Σ**_*H*_***V***_H_^*^ and obtain ${\hat{{\boldsymbol{F}}}}_{H}$ = ***U***_H_^*^Y_*H*_***V***_*H*_**Σ**_*H*_^(−1)^ as the exact DMD procedure. Thereafter, we perform eigendecomposition of ${\hat{{\boldsymbol{F}}}}_{H}\in {{\mathbb{C}}}^{p\times p}$ to obtain the set of eigenvalues *λ*_*j*_ and eigenvectors ***w***_*j*_ for *j* = 1, ..., *r*. A set of *λ*_*j*_ s approximates the Koopman eigenvalues. We call it row-type Hankel DMD. Conventional Hankel DMD modes are given by: ${\chi }_{j}={\lambda }_{j}^{(-1)}{{\boldsymbol{Y}}}_{H}{{\boldsymbol{Y}}}_{H}{{\boldsymbol{\Sigma }}}_{H}^{(-1)}{w}_{j}\in {{\mathbb{C}}}^{m\times 1}$ for *j* = 1, …, *p*. From the previous work^28^, the conventional Hankel DMD mode *χ*_*j*_ converges to the Koopman eigenfunctions ${\tilde{{\boldsymbol{\varphi }}}}_{j}$ if *m* is sufficiently large. We compute this row-type Hankel DMD for estimating Koopman eigenfunctions corresponding to one-dimensional dynamics.

For extracting the coordinative structure among observables (e.g. segmental angles), we have a problem that the conventional Hankel DMD modes ${\chi }_{j}\in {{\mathbb{C}}}^{m\times 1}$ (*j* = 1, …, *p*) cannot directly compute the coordinative structure between elements of a vector-valued observable (e.g. segmental angles). Then, we need to define row-type Hankel DMD modes regarding original observables (no duplication by delay embedding). Note that in general, we have several choices to define DMD modes (e.g. reviewed by the previous work^28^) in which the mode and the estimated value of Koopman eigenfunctions (here we denoted ${{\boldsymbol{\phi }}}_{j}\in {{\mathbb{C}}}^{m}$ and ${\psi }_{j}^{\ast }{{\boldsymbol{X}}}_{H1}\in {{\mathbb{C}}}^{nd}$, respectively) satisfy ${\psi }_{i}^{\ast }$*φ*_*j*_ = *δ*_*ij*_ (*δ*_*ij*_ is the Kronecker’s delta). Then, since conventional Hankel DMD mode *χ*_*j*_ is here used as eigenfunction ${\tilde{{\boldsymbol{\varphi }}}}_{j}$, we defined the complementary quantities, i.e. the row-type Hankel DMD modes, from the vector corresponding to the conventional eigenfunction value ${\psi }_{j}^{\ast }{{\boldsymbol{X}}}_{H1}\in {{\mathbb{C}}}^{nd}$ satisfying ***ψ***_i_^*^χ_*j*_ = *δ*_*ij*_. Finally, we selected the row-type Hankel DMD mode regarding original observables (without duplication) from ***ψ***_j_^*^X_*H*1_ by picking up the values corresponding to the initial value for each dimension.

The coordinative structure, can be computed in a more straightforward way as follows. We define the concatenated matrices as input matrix of Hankel DMD such that ***X***_*H*2_ = [***H***_1,1_, ***H***_2,1_, …, ***H***_*d*,1_]^T^ and ***Y***_*H*2_ = [***H***_1,2_, ***H***_2,2_, …, ***H***_*d*,2_]^T^. We call it column-type Hankel DMD (Fig. 1a), which is similar to the method such as used in the previous work^43^ with delay-coordinated matrices. For the above procedures, we can compute column-type Hankel DMD modes and eigenvalues in a similar procedure as exact DMD. However, column-type Hankel DMD modes duplicate by the delay embedding; thus, when we compare with basic DMDs and SVD-based method, we picked up the Hankel DMD modes without the delay for each dimension. Note that it is not straightforward for column-type Hankel DMD to estimate a one-dimensional eigenfunction for each dynamics from the viewpoint of phase model.

### Hankel DMD parameters and requirements

Here, we describe the procedure to determine Hankel DMD parameters and the theoretical requirements, to guarantee the validity for applying Hankel DMD to our data. For the computation of Hankel DMDs, we set the time length *n* to one gait cycle *T* frames, in order to compare the result of the previous works in walking intersegmental coordination^44,50^. In the previous work^28^, it was mentioned that a few hundred samples would be enough to determine the Koopman eigenvalues of periodic systems with high accuracy; thus, we fixed *n* = *T* and selected other parameters *p* and *m* as the following procedure.

For both types of Hankel DMDs, we need to determine the dimension *p* of truncated SVD and the dimension *m* of delay embedding. In this study, we determined *p* and *m* by considering both the convergence of Hankel DMDs as spectral analysis of a Koopman operator and the avoidance of fitting to high-frequency dynamics. In summary, we selected *p* = 50 and *m* = *T*, 2*T* for column- and row-type Hankel DMDs. For the actual procedure, see Supplementary Text S1. It should be noted that although we performed cross-validation widely used such as in statistics and machine learning area, it conflicted with the above criteria, which is shown as an independent numerical experiment in Supplementary Text S2 and Fig. S1.

### Statistical analyses

To assess the independent and combined effects of the walking speeds and various methods or modes, we tried to use two-way ANOVAs. However, the hypothesis of homogeneity of variances among methods or modes was rejected with Levene’s test, thus Friedman tests were performed for comparison among methods or modes. We used Wilcoxon’s sign rank test to compare the variables within the factor where a significant effect in Friedman test was found. The effect size was estimated using Kendall’s *W* for Friedman test and $r=z/\sqrt{N}$ for Wilcoxon’s sign rank test, where *z* is *z*-statistic estimated by Matlab function signrank. m and *N* is the number of samples. For all statistical calculations, *p* < 0.05 was considered significant. All statistical analyses were performed using the MATLAB 2018a Statistics and Machine Learning Toolbox (The MathWorks, Inc., MA, USA) and R-software package version 3.5.2.

## Supplementary information

Supplementary materials
