# Supplementary material for: Data-driven spectral analysis for coordinative structures in periodic human locomotion
Source: Sci Rep. 2019 Nov 14;9:16755. doi: 10.1038/s41598-019-53187-1 (PMC6856341; doi:10.1038/s41598-019-53187-1)
Supplement: Supplementary file 1 — Supplementary materials [file 41598_2019_53187_MOESM1_ESM.pdf]

Supplementary materials for:

Data-driven spectral analysis for coordinative structures in periodic human locomotion

Keisuke Fujii, Naoya Takeishi, Benio Kibushi, Motoki Kouzaki, Yoshinobu Kawahara

Text S1.

Embedding dimension of Hankel DMDs.

Here, we describe the procedure for the determination of embedding dimension of Hankel DMDs and quantitatively validated that Hankel DMDs are applicable to human walking data. First, from the viewpoint of the convergence[1], the dimension p of truncated SVD theoretically converges to the dimension of the Koopman invariant subspace k if $m \to \infty$. Practically, the previous work[1] used the hard threshold of SVD (1e-10). Another study in a similar algorithm[2] used the optimal hard threshold of SVD[3] when the noise level is unknown. Note that, obviously, the threshold of SVD is directly related to the dimension p of truncated SVD. For the dimension m of delay embedding, theoretically, sufficient m can approximate DMD modes to Koopman eigenfunctions (the necessary condition is obviously $m > k+1$)[1]. However, the effect of p (or the SVD threshold) and m on the convergence is not unclear in the actual locomotion data. Therefore, we examined various ps and ms to find the sufficient (not optimal) p and m using the validation dataset as a guide for the biological studies. Here, we used the convergence of the reconstruction error (defined above) for investigating the convergence of the estimation error of Koopman eigenvalues and modes.

Second, from the viewpoint of stably obtaining the desirable DMD results, we empirically know that Hankel DMDs with too large m and p generate eigenvalues with too high frequencies. This may cause the undesirable fitting to too high-frequency dynamics (i.e. DMD compute too large coefficients of the high-frequency dynamics) which should not be realistically considered. Thus, we visually selected certain m and p as small as possible while satisfying the condition in which the error is sufficiently small (here we call it *convergence* of the error below).

Then, we quantitatively validated that Hankel DMDs are applicable to human walking data. To compute both Hankel DMDs, we need to determine the dimension p of truncated SVD (or the number of Koopman eigenvalues) and the dimension m of delay embedding. Theoretically, Hankel DMDs with sufficient m obtain Koopman eigenvalues and eigenfunctions for a limit cycle[1]. We selected the dimensions m and p by considering the sufficiently small reconstruction error (we denote *convergence* here) and the avoidance of fitting to higher-frequency dynamics. First, from the perspective of the convergence, we investigated the sufficient m and p in Supplementary Fig. S13 online for three representational walking speeds of a participant. We used the convergence of the average reconstruction errors among participants to investigate the convergence of the estimation error of Koopman eigenvalues and modes. Overall, both column- and row-type Hankel DMDs with larger m and p converged to an error < 0.01 rad. For example, as a guide, if we select $p = 50$ and $m = T$ or $2T$ for column- and row-type Hankel DMDs (for T, see Supplementary Fig. S13a-c top online), the average error for all velocities and participants converged to 0.0013 ± 0.0006 and 0.0056 ± 0.0098 rad in column- and row-type Hankel DMDs, respectively.

Second, from the viewpoint of the avoidance of fitting to high-frequency dynamics, it may be better to select m and p as small as possible while satisfying the condition that the error converges. Therefore, we selected m and p as the above values ($p = 50$ and $m = T$ or $2T$). As a dynamical system to obey explicit governing equations, the results of similar convergence for the double pendulum (known frequency) in Fig. 2c and d, and walking model simulation data (unknown frequency similar to human data) are shown in Supplementary Fig. S2 online.

Text S2.

Cross-validation results of the dimensions of Hankel DMD.

For auxiliary information, it should be mentioned that there can be other approaches to determine m. In dynamical systems, determining delay embedding dimension has been discussed (e.g. reviewed by[4]) such as using false nearest neighbors[5] as used in the analysis of joint angles during locomotion[6]. However, these approaches can be directly applied to dynamical systems but may not be guaranteed to be suitable for DMDs. Among the approaches for nonlinear dynamical systems to fit the data to basis functions[7], the study by[8] used Akaike information criteria[9] after sparse identification. However, again, we did not choose m and p from the perspective of minimising the generalisation error; thus, we did not use any information criterion.

From the perspective of minimising generalisation errors, as an independent experiment of the main text, we also performed cross-validation to select p and m minimising the estimation error. Note that the Hankel DMD procedure does not estimate (or train) any parameter. Then, again, we divided data into validation and test datasets, and used the validation datasets for the leave-one-out cross-validation (LOOCV). Since p depends on m (column-type Hankel DMD: $p \geq \min(md,n)$ and row-type: $p \geq \min(m,n)$, we first determined m and then p to minimise the estimation error. However, we did not use the criteria to determine m and p, because too large m and p can decrease the generalisation errors but may cause the undesirable fitting to the too high frequencies.

Next, we show LOOCV results for various ms and ps using the box-plots.In Supplementary Fig. S1a and c, m in both Hankel DMDs decreased with the walking speed, whereas p in both Hankel DMDs in Supplementary Fig. S1b and d did not depend on the speed. These indicate that the selected ms in LOOCV seemed to be related to the above $m = T$ and the selected ps in LOOCV were larger than the above $p = 50$. The average LOOCV error for all velocities and participants was 0.0012 ± 0.0012 and 0.0015 ± 0.0007 rad in column- and row-type Hankel DMDs, respectively. For comparison with the above m and p, both average errors converged, but Supplementary Fig. S1b and d, some ps were too high and we considered that it may cause the undesirable fitting to the high-frequency dynamics. Therefore, we did not select m and p using cross-validation.

Text S3.

Companion-matrix DMD

In companion-matrix DMD[1,10], we form the companion matrix

$$\boldsymbol{C} = \begin{bmatrix} 0 & 0 & \dots & 0 & c_0 \\ 1 & 0 & \dots & 0 & c_1 \\ 0 & 1 & \dots & 0 & c_2 \\ \vdots & \vdots & \ddots & \vdots & \vdots \\ 0 & 0 & \dots & 1 & c_{\tau-1} \end{bmatrix}, \tag{1}$$

where $(c_0, c_1, \dots, c_{\tau-1})^T = \boldsymbol{X}^\dagger \boldsymbol{y}_\tau$. Then, we perform eigendecomposition of $\boldsymbol{C}$ to obtain the set of the eigenvalues λ_j and eigenvectors $\boldsymbol{w}_j$. Dynamic modes are given by $\boldsymbol{\psi}_j = \boldsymbol{X}\boldsymbol{w}_j$ for $j = 1, \dots, p$.

Text S4.

Conventional decomposition method based on SVD

The conventional intersegmental coordination (also called kinematic synergies) was extracted from the pre-processed elevation angle time-series matrix $\boldsymbol{\Theta} \in \mathbb{R}^{d \times \tau}$ ($d = 3, 4$ and τ is the time length) by SVD[11,12]. We call it SVD-based method in this study. The conventional two-dimensional coordinative structure (i.e. a plane) in three-dimensional angle space[13,14] can also be extracted by the same procedure. By applying SVD to the processed elevation angle matrix, the matrix was decomposed into the intersegmental coordination $\boldsymbol{z}_j$ and the temporal coordination $(\lambda_j \boldsymbol{v}_j)^{\mathrm{T}}$ such that

$$\hat{\boldsymbol{\Theta}}_p = \sum_{j=1}^{p} \boldsymbol{z}_j (\lambda_j \boldsymbol{v}_j)^T, \tag{2}$$

where p is the number of intersegmental coordination $(0 \geq p \geq d)$. Intersegmental coordination $\boldsymbol{z}_j$ represents the principal groups of the jth simultaneously active segmental group and temporal coordination $\lambda_j \boldsymbol{v}_j$ indicates the activation patterns of jth intersegmental coordination.

VAF is defined as the square error of the reconstructed data and the original data such that $\mathrm{VAF}_p = 1 - (\|\boldsymbol{\Theta} - \hat{\boldsymbol{\Theta}}_p\|_F^2)/\|\boldsymbol{\Theta}\|_F^2$, where $\|\cdot\|_F$ is the Frobenius norm. There are some procedures to determine the number of dimensions of the structure based on such as VAF, but for comparison with DMDs, we set $p = 2$ for the actual human locomotion and pendulum simulation data and $p = 3$ for the walking model simulation data. Moreover, in SVD-based method, since the augmented data dimension is variable in Hankel DMD, the absolute reconstruction error is defined as $(1/d\tau)\sum_{t=0}^{\tau-1}\sum_{j=1}^{p}\|\theta_{i,t} - \hat{\theta}_{i,t}\|$, where $\theta_{i,t}$ and $\hat{\theta}_{i,t}$ are scalar elements of $\boldsymbol{\Theta}$ and $\hat{\boldsymbol{\Theta}} = \sum_{j=1}^{p} \boldsymbol{z}_j (\lambda_j \boldsymbol{v}_j)^{\mathrm{T}}$ at dimension i and time t, respectively.

Text S5.

The details of double pendulum and walking model

Double pendulum simulation

As a validation of our approach using a physical phenomenon with a multi-link structure which obeys known ordinary differential equations with analytically-obtained frequency modes, we used a double pendulum model. Generally, the motion of a double pendulum is chaotic, but this study used a small initial condition and then the equation can be linearised and obtained two eigenfrequencies. For simplicity, we considered a double pendulum consisting of two pendulums with the same length l and weight m attached end to end, as shown in Fig. 2c. Parameters θ_1 and θ_2 are also in Fig. 2c (we set $m = 1$ kg and $l = 1$ m).

The governing equations are as follows:

$$\begin{aligned}\frac{d^2\theta_1}{dt} &= \frac{g(-2\sin(\theta_1)+\sin(\theta_2)\cos(\theta_1-\theta_2))-l(\dot{\theta}_2^2-\dot{\theta}_1^2\cos(\theta_1-\theta_2))\sin(\theta_1-\theta_2)}{l(2-\cos(\theta_1-\theta_2)^2)},\\ \frac{d^2\theta_2}{dt} &= \frac{2g(\cos(\theta_1-\theta_2)\sin(\theta_1)-\sin(\theta_2))+l(2\dot{\theta}_1^2+\dot{\theta}_2^2\cos(\theta_1-\theta_2))\sin(\theta_1-\theta_2)}{l(2-\cos(\theta_1-\theta_2)^2)}.\end{aligned}$$

For small initial conditions (we set $\theta_1 = \theta_2 = \pi/8$ and $\dot{\theta}_1 = \dot{\theta}_2 = 0$), we obtain approximated linear systems

$$\begin{aligned}\frac{d^2\theta_1}{dt} &\approx \frac{g(-2\theta_1+\theta_2)}{l},\\ \frac{d^2\theta_2}{dt} &\approx \frac{2g(\theta_1-\theta_2)}{l}.\end{aligned}$$

By solving the secular equation, we analytically obtain two eigenfrequencies $\sqrt{2\pm\sqrt{2}}\sqrt{g/l}$. We verified our approach using the simple model with explicit governing equation and frequency modes. In the simulation, the time step and the duration were set to $1/20$ s and 500 s in total. We set the analyzed interval to 160 time points based on Fig. 2d (here, we did not use the information of the true frequencies). Similarly to the actual locomotion data, we divided all data into a validation dataset (10 sequences) for determining the parameters of Hankel DMDs and a test dataset (10 sequences) for the remaining analyses.

Walking model and simulation

Next, to understand the equations of motion in locomotion and to verify our approach using a model simulation data with explicit governing equation (without noise) but with unknown frequency modes, we describe a simple human multi-link model. First, we describe a general overview of the dynamics, and indicate that the gait dynamics can be partly approximated to be a nonlinear function of the segmental angles $\boldsymbol{\theta}$. The dynamics of a multi-link model are derived in the following Lagrangian equations:

$$\boldsymbol{D}(\boldsymbol{\theta})\ddot{\boldsymbol{\theta}}+\boldsymbol{H}(\boldsymbol{\theta})\dot{\boldsymbol{\theta}}+\boldsymbol{G}=\boldsymbol{T}, \tag{3}$$

where $\boldsymbol{D}(\boldsymbol{\theta})$ is a positive definite and symmetric inertia matrix, $\boldsymbol{H}(\boldsymbol{\theta})$ is the matrix of a centrifugal and Coriolis terms, $\boldsymbol{G}(\boldsymbol{\theta})$, $\boldsymbol{\theta}$, $\dot{\boldsymbol{\theta}}$, $\ddot{\boldsymbol{\theta}}$ are a gravity term, generalised coordinates, velocities and accelerations, respectively (more details can be found such as in[15]). $\boldsymbol{T}$ is a generalised torque term, that has been complicatedly modeled in general but precisely unknown in actual humans.

Next, Eq (3) can be transformed into the following form:

$$\frac{d\dot{\boldsymbol{\theta}}}{dt} = \boldsymbol{f}(\boldsymbol{\theta})+\boldsymbol{g}(\boldsymbol{\theta},\dot{\boldsymbol{\theta}}), \tag{4}$$

where $\boldsymbol{f}(\boldsymbol{\theta}) = \boldsymbol{D}(\boldsymbol{\theta})^{-1}(\boldsymbol{T} - \boldsymbol{G}(\boldsymbol{\theta}))$ and $\boldsymbol{g}(\boldsymbol{\theta}, \dot{\boldsymbol{\theta}}) = -\boldsymbol{D}(\boldsymbol{\theta})^{-1}\boldsymbol{H}(\boldsymbol{\theta})\dot{\boldsymbol{\theta}}^2$. The results of previous work[16] in the numerical simulation showed that $\|\boldsymbol{f}(\boldsymbol{\theta})\| \gg \|\boldsymbol{g}(\boldsymbol{\theta}, \dot{\boldsymbol{\theta}})\|$. In other words, $\boldsymbol{f}(\boldsymbol{\theta})$ represents the absolute domination in the dynamics of the biped model. Therefore, gait dynamics can be approximately represented by function $\boldsymbol{f}(\boldsymbol{\theta}) = \boldsymbol{D}(\boldsymbol{\theta})^{-1}(\boldsymbol{T} - \boldsymbol{G}(\boldsymbol{\theta}))$ along the phase portrait of $\boldsymbol{\theta}$. Hence, segmental angles are selected as the kinematic parameters, because the gait dynamics can be partly approximated to be a nonlinear function of the segmental angles, except for the torque term.

Next, as a walking model simulation for comparison with the actual walking data, we used a well-known five-link neural oscillator control model[15] for simplicity (again, here we used four angles: left and right thigh and shank angles). We used the cited body parameters and motion equations and set the constant input as 6. In the simulation, the time step and the duration were set to 10^{-6} s and 120 s in total, but thereafter we employed the down-sampling into 100 Hz for the computation of DMDs. We set the analyzed interval to a gait cycle similarly to the actual human locomotion. We divided all data into validation datasets (10 sequences) for determining the parameters of Hankel DMD and test datasets (10 sequences) for the remaining analyses. Data is deposited on Dryad (doi: https://doi.org/10.5061/dryad.8421659/3).

References

1. Arbabi, H. & Mezić, I. Ergodic theory, dynamic mode decomposition, and computation of spectral properties of the koopman operator. *SIAM J. on Appl. Dyn. Syst.* **16**, 2096–2126 (2017).
2. Brunton, S. L., Brunton, B. W., Proctor, J. L., Kaiser, E. & Kutz, J. N. Chaos as an intermittently forced linear system. *Nat. Commun.* **8**, 19 (2017).
3. Gavish, M. & Donoho, D. L. The optimal hard threshold for singular values is $4/\sqrt{3}$. *IEEE Transactions on Inf. Theory* **60**, 5040–5053 (2014).
4. Bradley, E. & Kantz, H. Nonlinear time-series analysis revisited. *Chaos: An Interdiscip. J. Nonlinear Sci.* **25**, 097610 (2015).
5. Kennel, M. B., Brown, R. & Abarbanel, H. D. Determining embedding dimension for phase-space reconstruction using a geometrical construction. *Phys. Rev. A* **45**, 3403 (1992).
6. Nessler, J. A., De Leone, C. J. & Gilliland, S. Nonlinear time series analysis of knee and ankle kinematics during side by side treadmill walking. *Chaos: An Interdiscip. J. Nonlinear Sci.* **19**, 026104 (2009).
7. Brunton, S. L., Proctor, J. L. & Kutz, J. N. Discovering governing equations from data by sparse identification of nonlinear dynamical systems. *Proc. Natl. Acad. Sci.* **201517384** (2016).
8. Mangan, N., Kutz, J., Brunton, S. & Proctor, J. Model selection for dynamical systems via sparse regression and information criteria. *Proc. Royal Soc. A: Math. Phys. Eng. Sci.* **473** (2017).
9. Akaike, H. A new look at the statistical model identification. *IEEE Transactions on Autom. Control.* **19**, 716–723 (1974).
10. Rowley, C. W., Mezić, I., Bagheri, S., Schlatter, P. & Henningson, D. S. Spectral analysis of nonlinear flows. *J. Fluid Mech.* **641**, 115–127 (2009).
11. Funato, T., Aoi, S., Oshima, H. & Tsuchiya, K. Variant and invariant patterns embedded in human locomotion through whole body kinematic coordination. *Exp. Brain Res.* **205**, 497–511 (2010).
12. Funato, T., Aoi, S., Tomita, N. & Tsuchiya, K. A system model that focuses on kinematic synergy for understanding human control structure. In *IEEE International Conference on Robotics and Biomimetics (ROBIO'12)*, 378–383 (IEEE, 2012).
13. Borghese, N. A., Bianchi, L. & Lacquaniti, F. Kinematic determinants of human locomotion. *The J. Physiol.* **494**, 863–879 (1996).
14. Lacquaniti, F., Grasso, R. & Zago, M. Motor patterns in walking. *Physiology* **14**, 168–174 (1999).
15. Taga, G., Yamaguchi, Y. & Shimizu, H. Self-organized control of bipedal locomotion by neural oscillators in unpredictable environment. *Biol. Cybern.* **65**, 147–159 (1991).
16. Deng, M., Wang, C., Cheng, F. & Zeng, W. Fusion of spatial-temporal and kinematic features for gait recognition with deterministic learning. *Pattern Recognit.* **67**, 186–200 (2017).
17. Kutz, J. N., Brunton, S. L., Brunton, B. W. & Proctor, J. L. *Dynamic Mode Decomposition: Data-Driven Modeling of Complex Systems* (SIAM, 2016).

Supplementary Figures

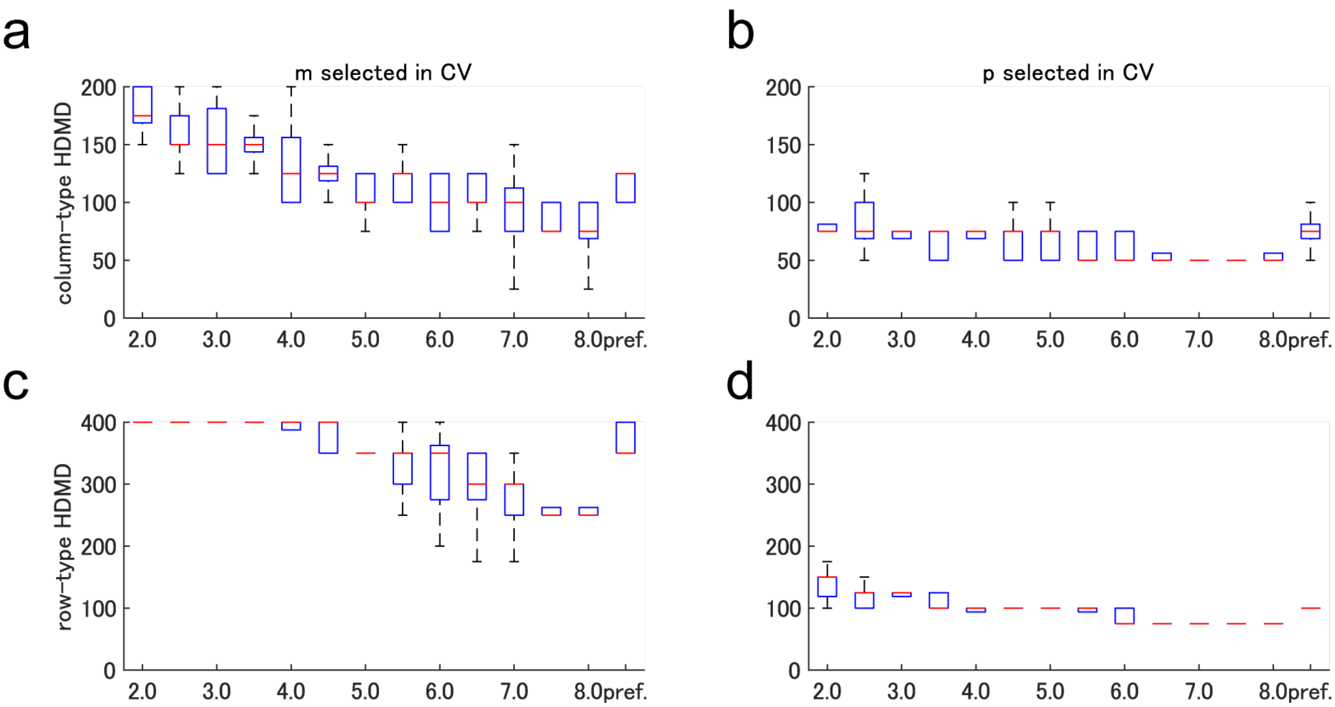

Fig. S1. **Selected dimensions** m **and** p **in cross validation.** Boxplots of selected dimensions m (a and c) and p (b and d) for column-type (a and b) and row-type (c and d) Hankel DMDs during various walking velocities are shown. The central mark (red) indicates the median, and the bottom and top edges of the box indicate the 25th and 75th percentiles, respectively. The whiskers extend to the most extreme data points.

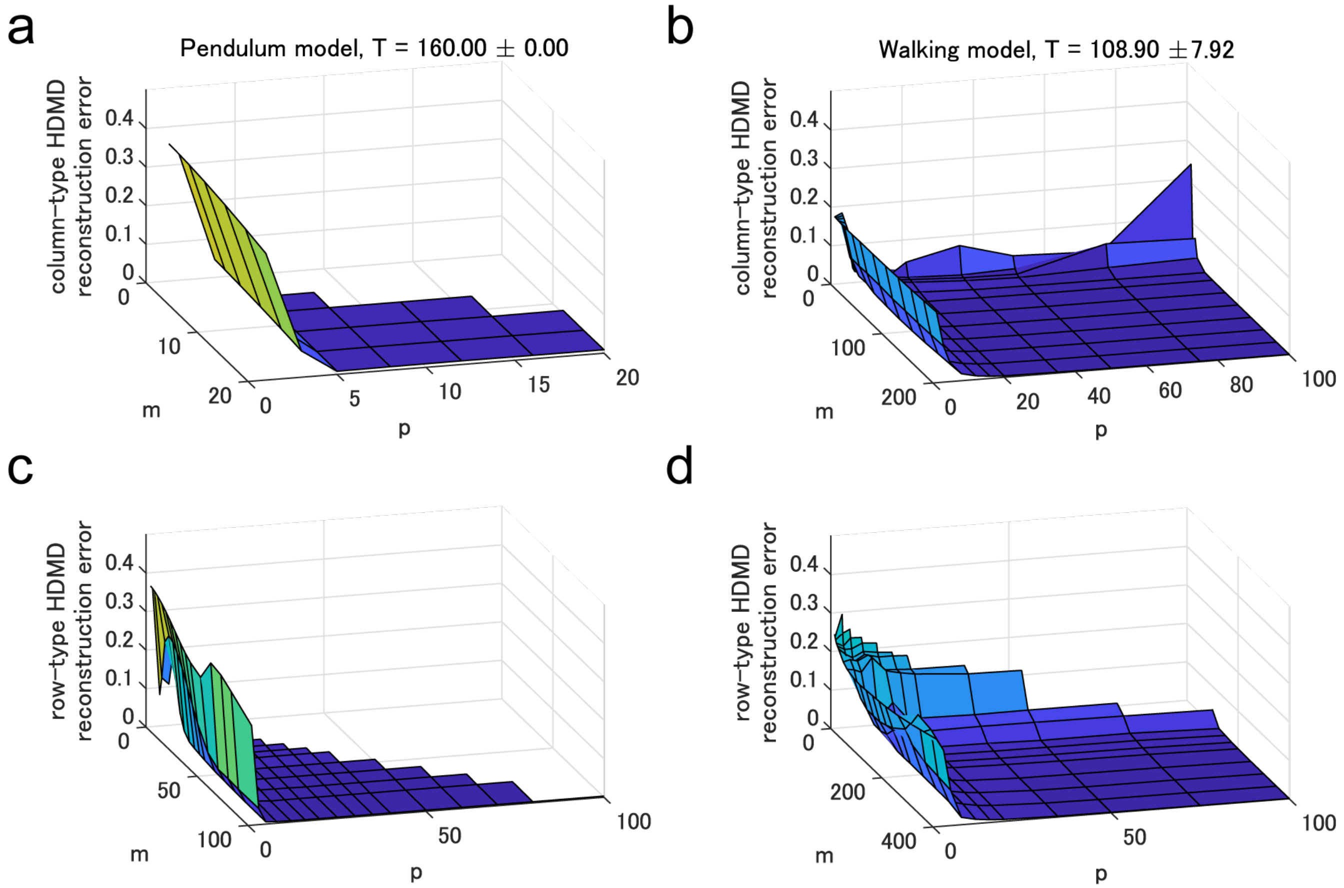

Fig. S2. Convergence of Hankel DMDs for two simulation data. Examples of the reconstruction error for various m and p for column-type (a and b) and row-type (c and d) Hankel DMDs for the simulation data of the pendulum model and walking model are shown. Similarly to the human walking data, both column- and row-type Hankel DMDs with larger m and p converged to a certain error. We selected $p = 20$ and $m = 20, 100$ for column- and row-type Hankel DMDs for the pendulum model (error in column-type: 0.0090 ± 0.0047 rad, error in row-type: 0.0031 ± 0.0004 rad) and $p = 50$ and $m = T, 2T$ for the walking model (error in column-type: 0.0021 ± 0.0013 rad, error in row-type: 0.0073 ± 0.0102 rad). For the pendulum model, we set m to 20 and 100 in the column-type and row-type because of the rank deficiency and the computation of the Koopman eigenfunction, respectively. The other criteria were the same as those described in the main text. The analyzed interval (n) is shown in (a and b) top.

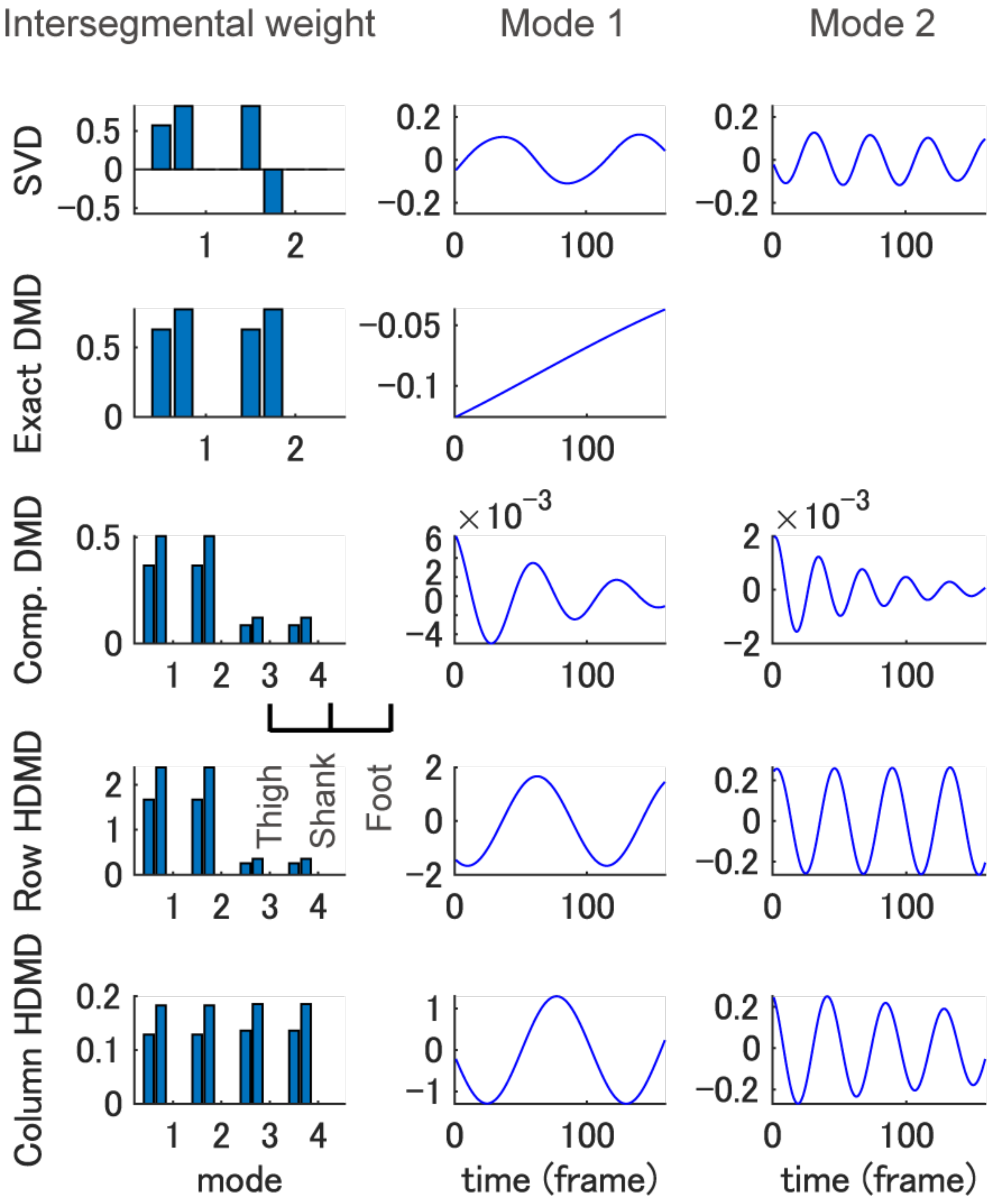

Fig. S3. Decomposition of segmental angles of double pendulum simulation. Examples of decomposition results into intersegmental weights (or DMD modes) and time dynamics by various methods for double pendulum simulation are shown. Configurations are the same as in Fig. 3, but we indicate two dominant modes because of the property of the system. Exact DMD and companion-matrix DMD show incorrect decomposition because of the smaller data dimension $d = 2$ (this is a well-known problem explained such as in[17]). Reconstruction error of the dominant modes is lower for SVD-based method ($< 10^{-15}$ rad), row-type Hankel DMD (0.0160 ± 0.0020 rad), row-type Hankel DMD (0.0274 ± 0.0027 rad), companion-matrix DMD (0.2566 ± 0.0050 rad) and exact DMD (0.2663 ± 0.0216 rad) in this order.

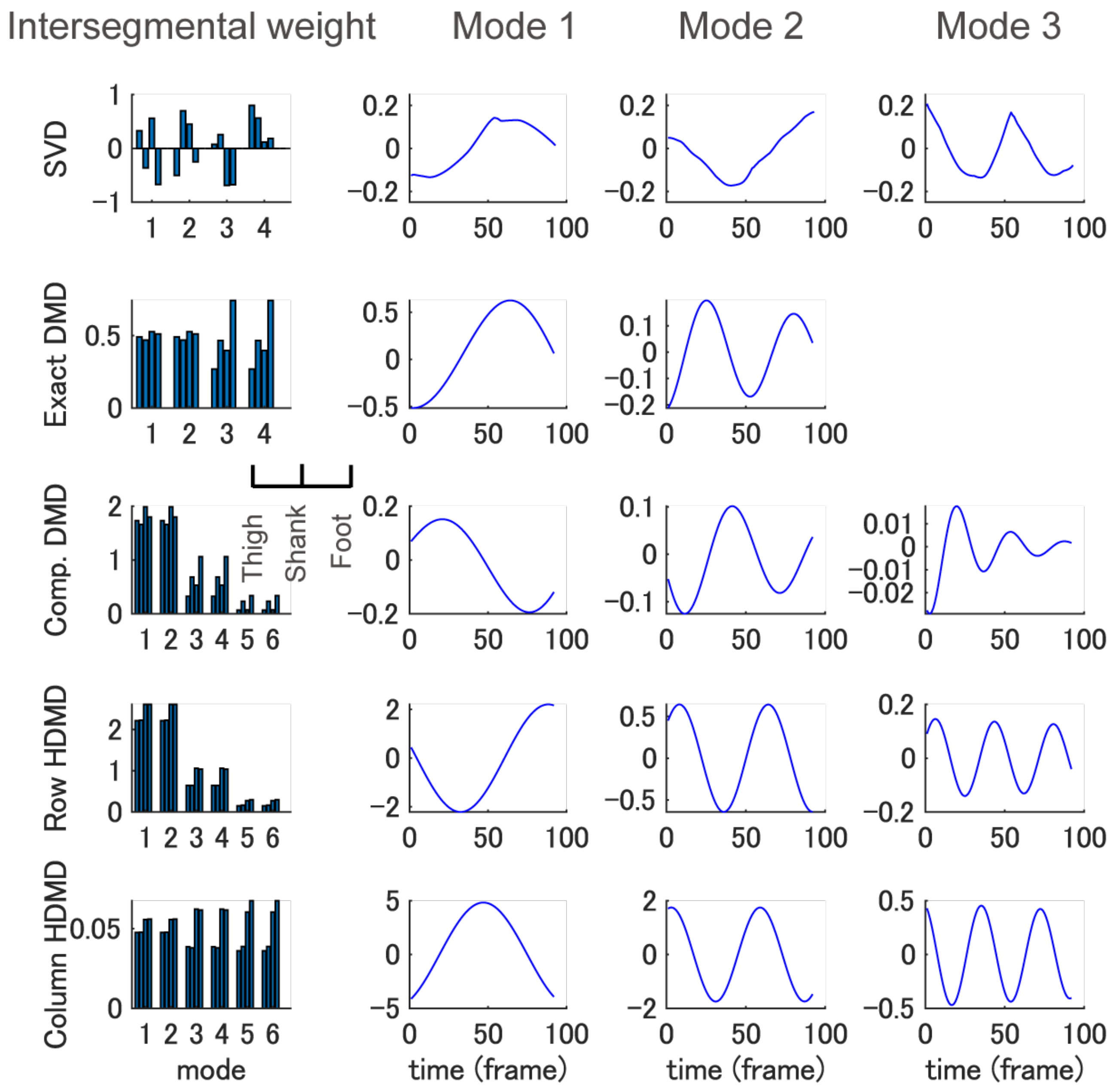

Fig. S4. Decomposition of segmental angles of walking model simulation. Examples of decomposition results into intersegmental weights (or DMD modes) and time dynamics by various methods for double pendulum simulation are shown. Configurations are the same as in Fig. 3. Results were similar to Fig. 3. Reconstruction error of the dominant modes are lower for SVD-based method ($< 10^{-15}$), column-type Hankel DMD (0.0354 ± 0.0001 rad), row-type Hankel DMD (0.0373 ± 0.0008 rad), companion-matrix DMD (0.1356 ± 0.0348 rad) and exact DMD (0.1411 ± 0.0043 rad) in this order.

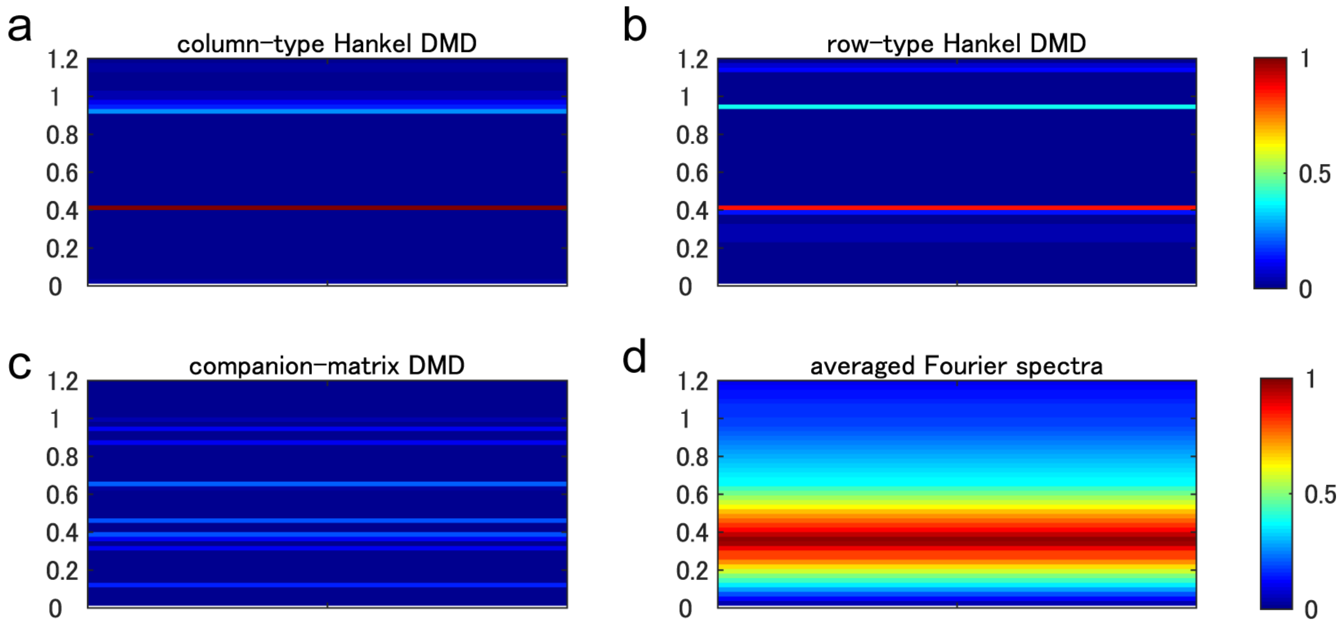

Fig. S5. DMD eigenvalues of double pendulum simulation. Temporal frequency spectra averaged among all participants in frequency domain to the gait frequency for (a) column-type and (b) row-type Hankel DMD, (c) companion-matrix DMD and (d) Fourier transformation for the double pendulum simulation data are shown. Configurations are the same as Fig. 6 for clarity, but the horizontal axis indicates only one condition. The spectrum of each sequence was normalised so that the maximal norm is 1. Column- and row-type Hankel DMD seemed to obtain two eigenfrequencies (0.3815 and 0.9211 Hz). However, companion-matrix DMD did not distinctly indicate the eigenfrequencies. Averaged Fourier spectra were seemingly smoothed around only one eigenfrequency.

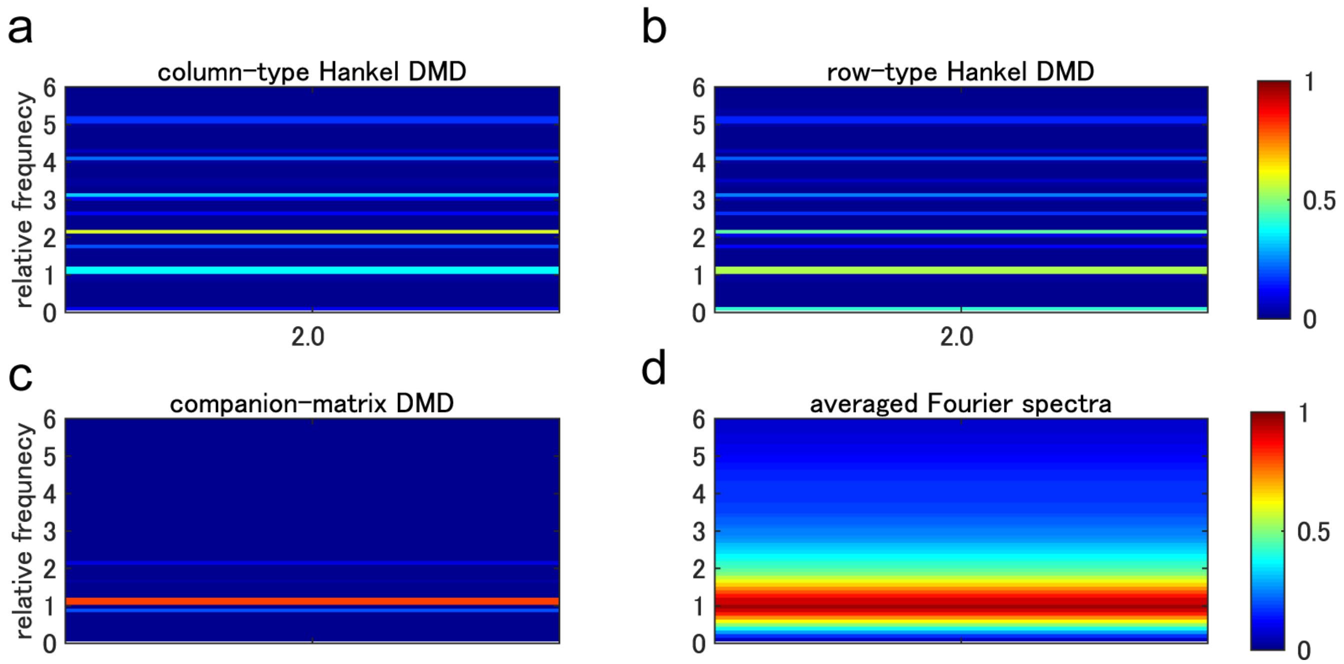

Fig. S6. DMD eigenvalues of walking model simulation. Temporal frequency spectra averaged among all participants in frequency domain to the gait frequency for (a) column-type and (b) row-type Hankel DMDs, (c) companion-matrix DMD and (d) Fourier transformation for the walking model simulation data are shown. Configurations are the same as in Fig. 6 for clarity, but the horizontal axis indicates only one condition. Results were similar to those in Fig. 6.

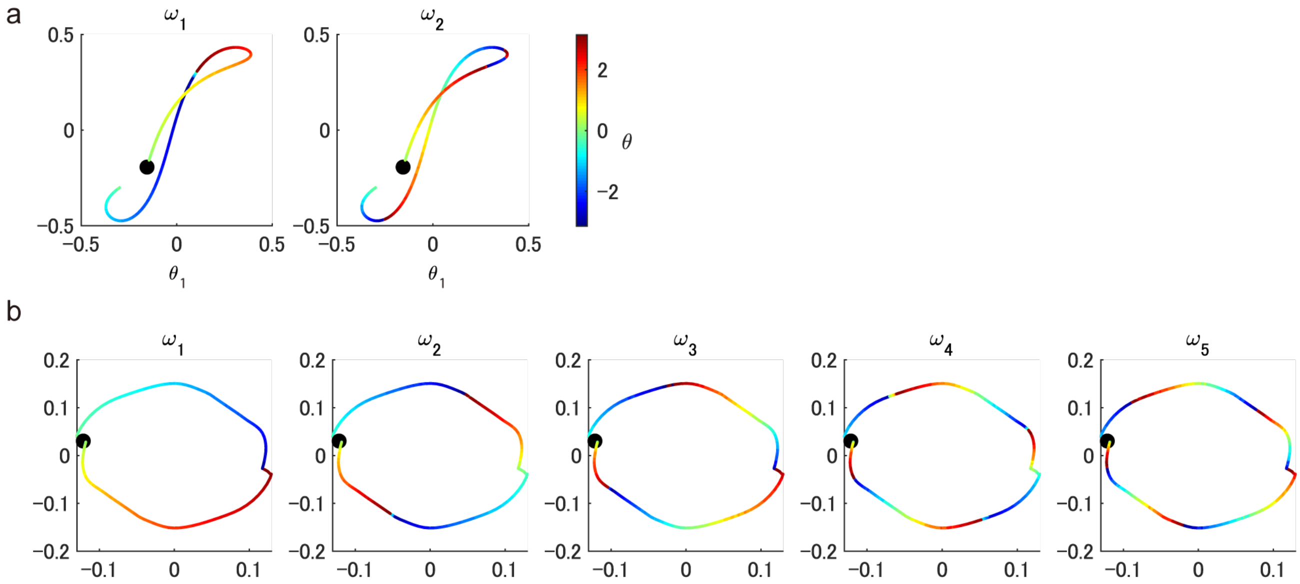

Fig. S7. Phases of Koopman eigenfunctions for two simulation data. Phases computed by the argument of the Koopman eigenfunctions estimated by row-type Hankel DMD are shown for (a) the double pendulum simulation and (b) each walking speed and five harmonic frequencies. (a) For the pendulum simulation, because of the two-dimensional data, we visualise the phase on the two-dimensional trajectories. (b) For the walking simulation data, similarly to the human walking data, we visualise the phase on the two-dimensional structure obtained by the conventional SVD-based method. For clarity, we aligned the initial phases to near-zero values by the time-shift for all eigenfunctions. All trajectories start from the black dots.

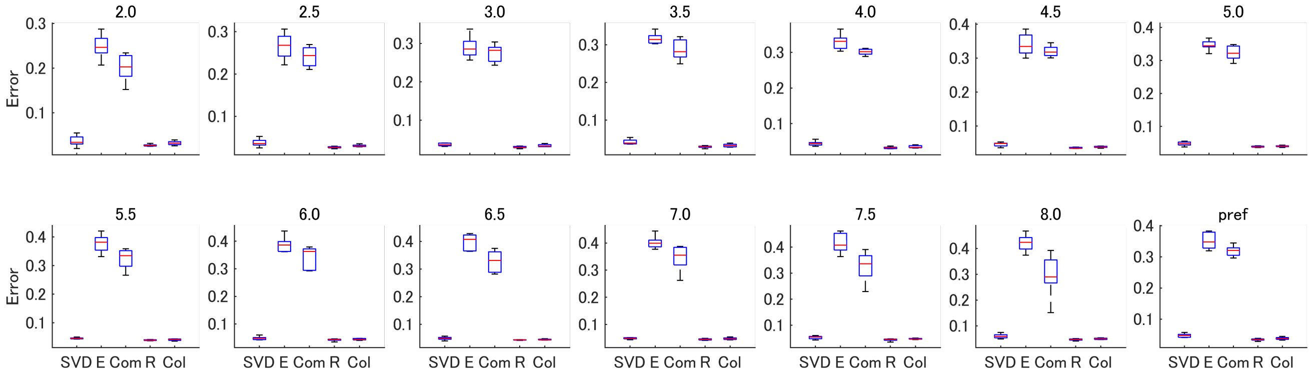

Fig. S8. Reconstruction error data. Reconstruction error of five methods averaged within walking participants for all 14 velocities are shown. SVD, E, Com, R, Col indicate SVD-based method, exact DMD, companion-matrix DMD, row-type and column-type Hankel DMD, respectively.

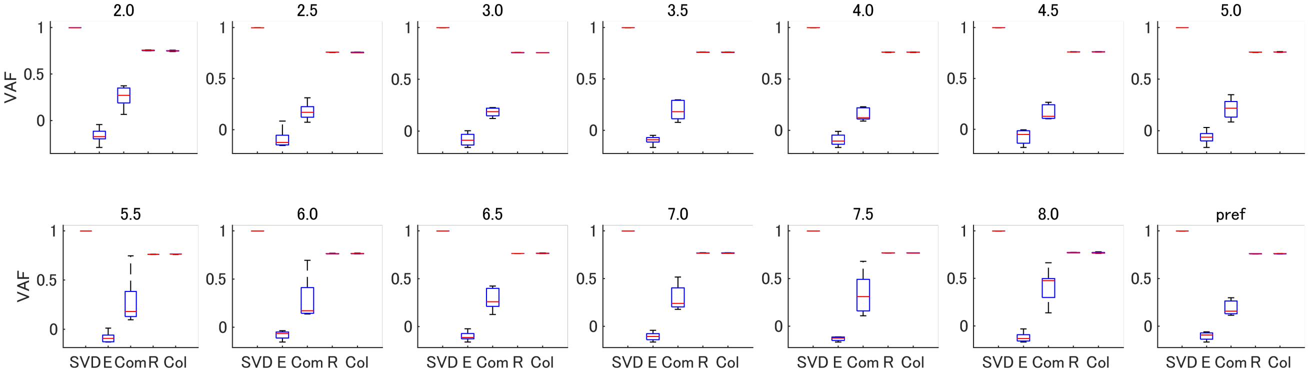

Fig. S9 VAF data. VAF of five methods averaged within walking participants for all 14 velocities are shown. SVD, E, Com, R, Col indicate SVD-based method, exact DMD, companion-matrix DMD, row-type and column-type Hankel DMD, respectively.

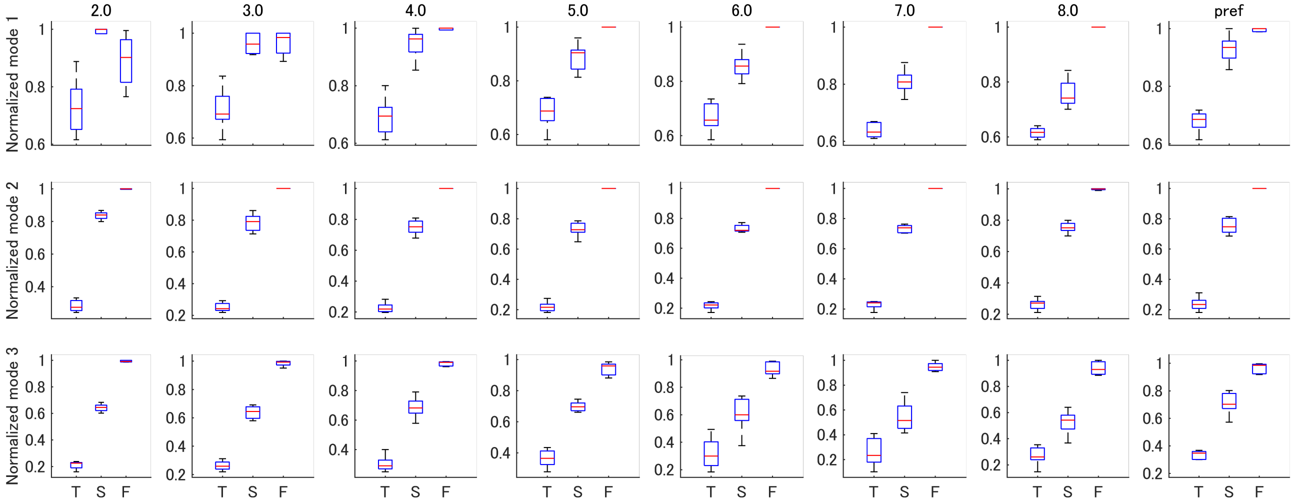

Fig. S10. Boxplot of column-type Hankel DMD Modes. Normalised column-type Hankel DMD Mode 1 to 3 averaged within walking participants for eight velocities are shown. T, S and F indicate thigh, shank and foot angles, respectively. The tendency was similar to that of row-type Hankel DMD modes.

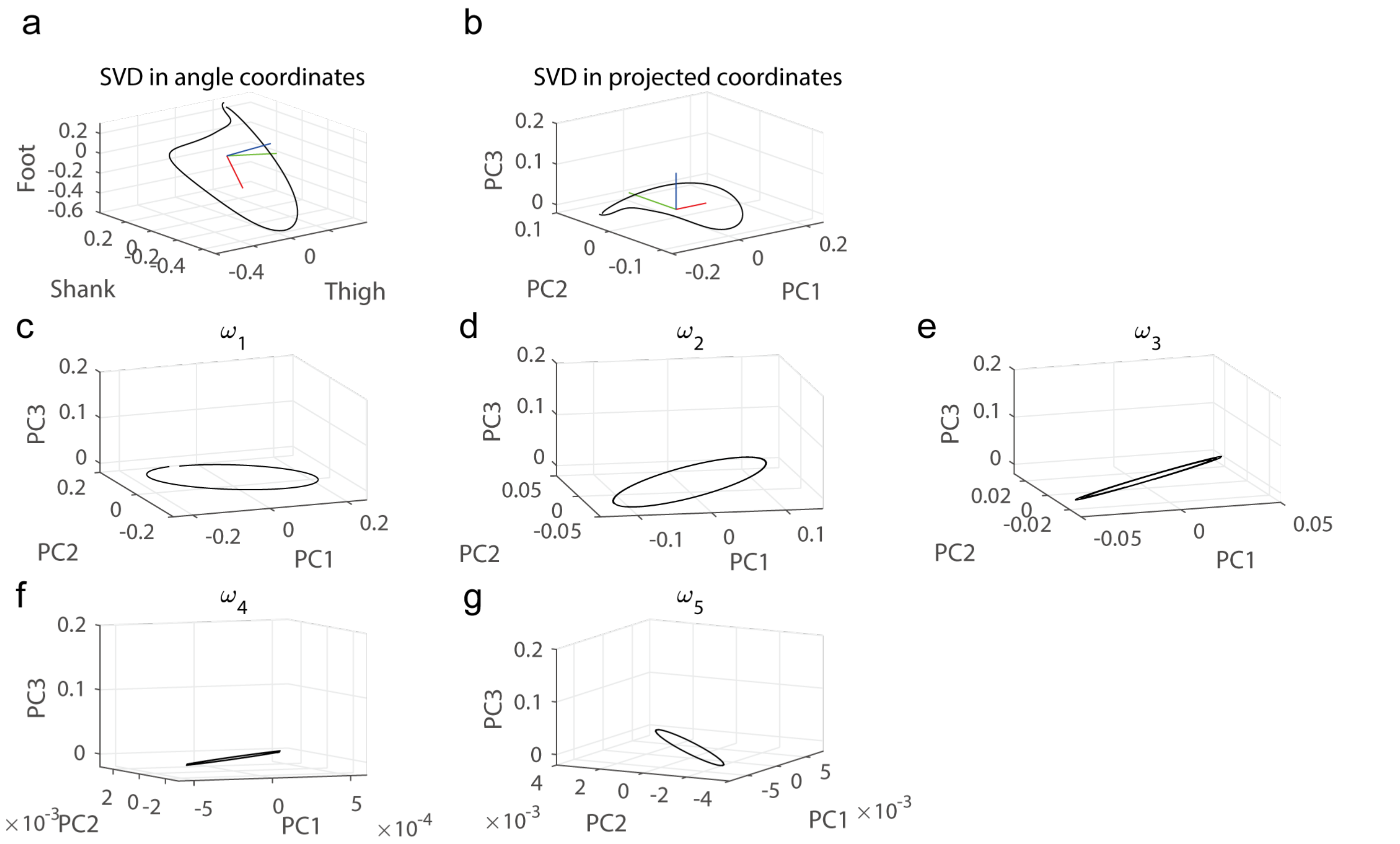

Fig. S11. Relation to the existing coordinative structure. (a) The trajectory in three angle coordinates with the project coordinates (red, green and blue indicate the first, second and third directions of principal components (PC1, PC2 and PC3) computed by existing SVD-based method. (b) The trajectory in projected coordinates by rotating (a). (c-g) Trajectories of five harmonic frequencies in the projected coordinates extracted by row-type Hankel DMD.

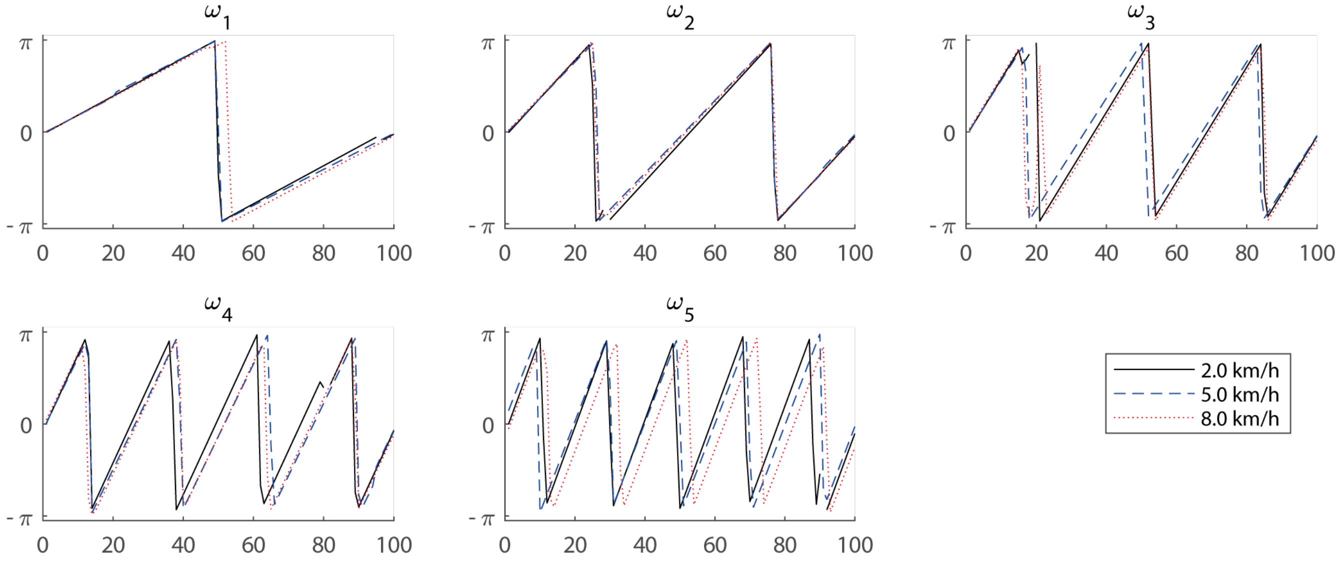

Fig. S12. Normalised time-series of the phases of Koopman eigenfunctions. Phases computed by the argument of the Koopman eigenfunctions estimated by row-type Hankel DMD are shown for three walking speeds and five harmonic frequencies (a-e). Time series were normalised to 100 time stamps for indicating temporal normalisation seems to be delayed the phase at higher walking speed (but actually advanced because of the higher gait frequency or shorter gait cycle as shown in Fig. 7). For clarity, we aligned the initial phases to near-zero values by the time-shift for all eigenfunctions (non-continuous sequences are the byproduct of the time-shift).

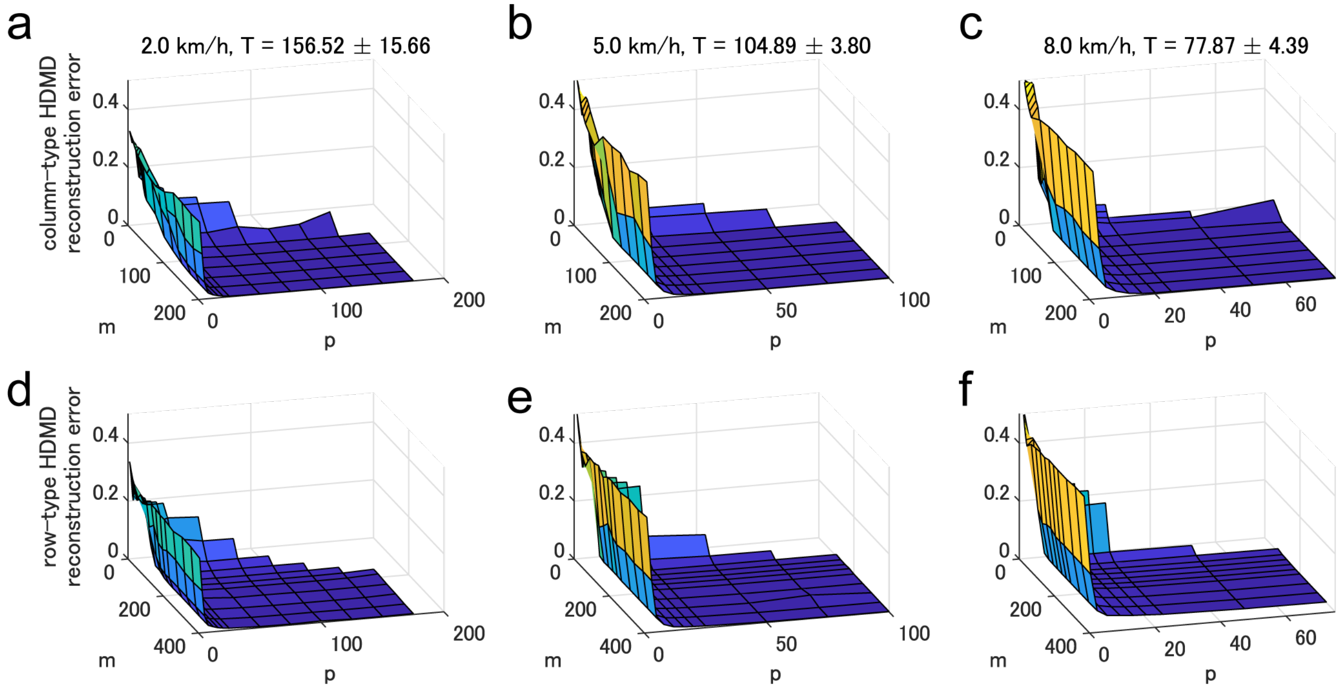

Fig. S13. Convergence of Hankel DMDs. Examples of the reconstruction error for various m and p for column-type (a-c) and row-type (d-f) Hankel DMDs during 2.0 km/h (a and d), 5.0 km/h (b and e) and 8.0 km/h (c and f) walk are shown. Overall, both column- and row-type Hankel DMDs with larger m and p converged to a certain error. In particular, the effect of m seemed to depend on walking speed (i.e. a higher speed or shorter gait cycle converged faster with an increase of m), but that of p did not seem to be independent of the walking speed if m is sufficient. We selected $p = 50$ and $m = T, 2T$ for column- and row-type Hankel DMDs. One gait cycle for each velocity is shown in (a-c) top.
